# A Water‐Triggered Sensor for Self‐Powered Flood Alarming

**DOI:** 10.1002/advs.202503815

**Published:** 2025-07-09

**Authors:** Mingqi Zhao, Shanzhi Lyu, Yingchao Ma, Nan Zhang, Yapei Wang

**Affiliations:** ^1^ School of Chemistry and Life Resources Renmin University of China Beijing 100872 China; ^2^ Department of Energy and Power Engineering Tsinghua University Beijing 100082 China

**Keywords:** flood alarming system, hydrogel, self‐powered sensor

## Abstract

Devastating natural disasters are prompting the need for effective early warning systems. The warning systems relying on cables or batteries are much expensive and not suitable for low‐lying areas or remote environments. This research proposes a water‐triggered sensor that has no energy consumption in dry conditions while becoming activated in the presence of water. The sensor is self‐powered by a switchable galvanic battery which is made of two metallic electrodes isolated by an ion‐loaded xerogel. When the battery is subjected to a water environment, the rapid hydration of the xerogel arouses the redox reaction between two electrodes, subsequently outputting the electrical power to trigger the sensing system associated with timely signal generation and transmission. To ensure adequate stability and durability against the ambient humidity variation, the battery is sealed with a polymer coat which is inert to the moisture but permeable to water. Since the sensor can be built on a paper base, it is suitable for Miura origami which is beneficial for improving the output power and minimizing the sensor size. Outdoor experiments confirm that the sensor is able to rapidly discriminate floods and warn nearby people and remote‐control centers.

## Graphical abstract

Urban floods related to global warming generally cause significant casualties. Traditional cable flood warning systems are costly, while wireless ones face power limitations. This research proposes a self‐powered flood sensor with a water‐triggered Galvanic battery, enabling rapid flood warnings with great promise of low cost and long service lifetime.

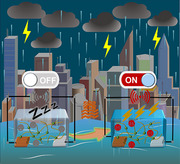

## 1 Introduction

**Scheme 1:**
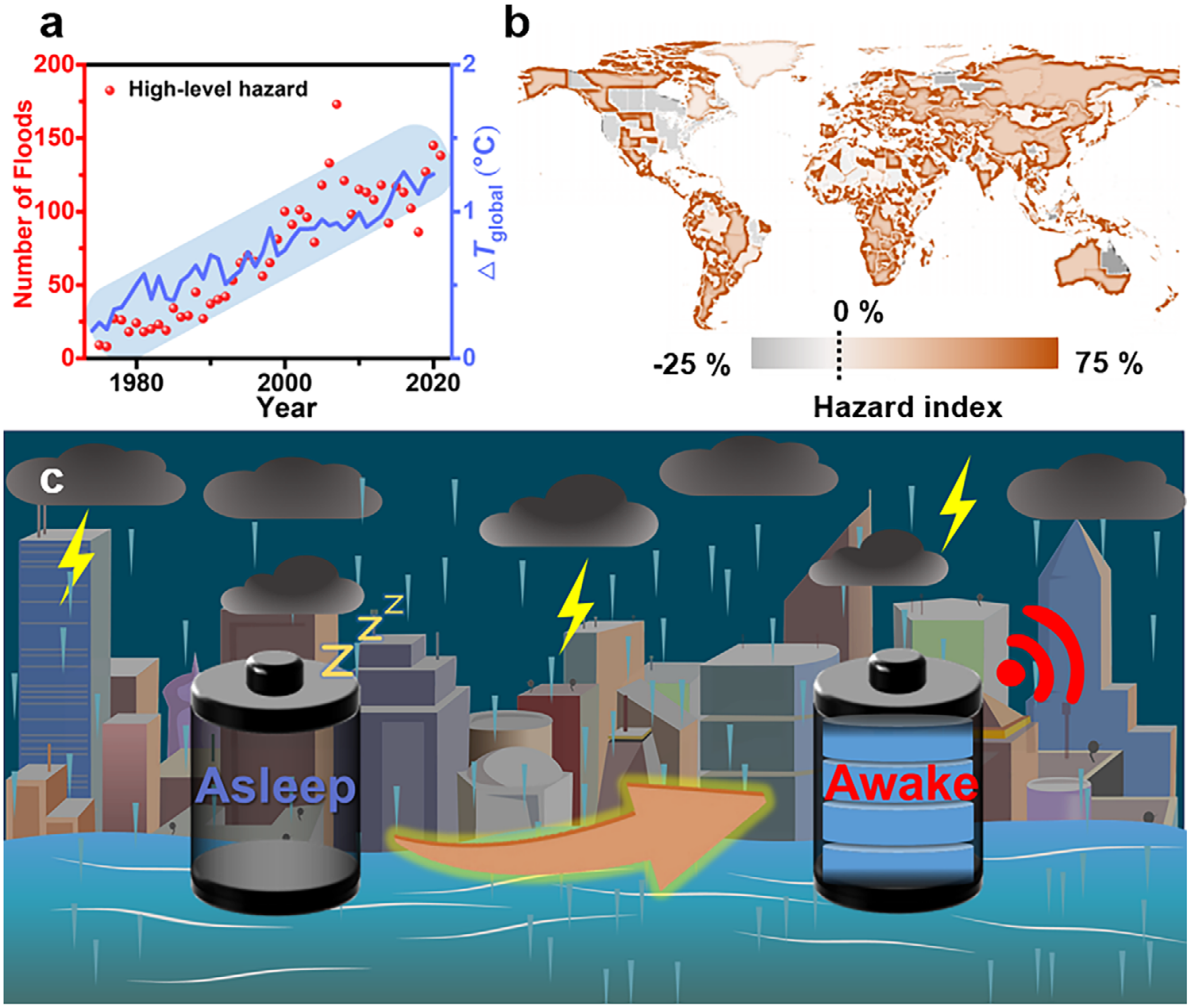
Global flood hazard and the early‐term flood warning sensors. a) The relationship between the number of global major floods (red dots) and the global temperature change (blue line) over the past 40 years. b). Hazard index caused by urbanization of possible floods in different regions over the past decades. The hazard index represents the likelihood of the region after urbanization experiencing significant flood disasters compared to fifty years ago. c) Schematic of the self‐powered early‐term flood warning sensor.

From the accounts in the Old Testament of the Bible to the Great Yu in the East, the struggle against flooding has been lasting throughout the development of human civilization. Publicly available statistics indicate that flood disasters have always been among the primary natural disasters in recent years (As shown in Table S1, Supporting Information).^[^ ^1^ ^]^ What's worse, the intensifying greenhouse effect due to global warming is raising the global average temperature by 1.5 °C (**Scheme** **1**a, blue line).^[^ ^2^ ^]^ This is the primary reason why the number of major global floods has risen by ≈700% over the past forty years (Scheme 1a, red dots).^[^ ^3^, ^4^ ^]^ Besides, the acceleration of global urbanization leads to an increasing number of areas facing a higher risk of flooding over the world (Scheme 1b).^[^ ^5^ ^]^ Statistical data demonstrates that floods are characterized by extensive hazardous areas and severe impacts, resulting in significant human casualties and economic losses in recent years,^[^ ^6^ ^]^ in either developed or developing countries (**Table** **1**).^[^ ^7^ ^]^ For example, the flood in Derna, Libya in September 2023 resulted 11 000 deaths as the rainfall far exceeded the warning limits of the drainage system and wasn't undetected in time. Similarly, in 2024, the heavy rainfall associated with Hurricane Helene caused a rapid raising in river level, giving rise to extensive property damage and at least 236 fatalities in Dixie in the USA.

**Table 1:** An overview of the major flood disasters over the world in recent years.

City	Date	Reason	Economic loss [$]	Affected people	Dead people	Disaster area[km^2^]
Chennai	2015.11	Monsoon	13 billion	>4 million	>500	> 92000
Macau	2017.08	Storm	1.56 billion	–	16	10.76
Niger	2018.08	Storm	100 billion	3 million	28	257400
Hiroshima	2018.07	Monsoon	2.8 billion	1.5 million	220	109670
Zhengzhou	2021.07	Typhoon	5.9 billion	3 million	292	527300
Derna	2023.09	Storm	1.65 billion	1.5 million	>1100	5000
Dubai	2024.04	Storm	>10 billion	–	19	32.7
Meizhou	2024.06	Storm	0.3 billion	164634	38	2000
Shangzuo	2024.07	Storm	0.3 billion	212000	62	6500
Kangding	2024.08	Storm	–	1000	27	194
Khartoum	2024.08	Storm	–	156300‐	114	–
Shangqiu	2024.09	Storm	–	433136	–	6960
Dixie	2024.09	Typhoon	110 billion	3100000	237	–
Valencia	2024.1	Storm	11.4 billion	190000	233	500
Batangas	2024.1	Typhoon	0.3 billion	9620000	160	6300

Flood resulting from heavy rain is generally predicted by weather forecasting according to satellite observation, yet it is difficult to specify the areas where flood may be formed suddenly. Floods are characterized with rapid evolution and unpredictable pathways in low‐lying areas, including underpasses, subway stations, and villages at the foot of mountains which account for ≈70% of the casualties in flood disasters. The need to early warn the flood formation and minimize its destructive effect has spurred intense research in establishing flood monitoring systems.^[^ ^8^ ^]^ Traditional monitoring ways based on manual patrols are limited by high costs and possible omissions. Installation of cable warning systems in low‐lying areas can offer greater safety and precision for early flood warnings, whereas they come with extremely expensive installation and maintenance besides the possible malfunction due to electrical leakage in flood events. Moreover, the cable warning systems require time‐consuming and labor‐intensive reconstruction after each flood flush. These disadvantages make it difficult to achieve real‐time monitoring in low‐lying areas of urban and suburb regions via cable flood warning systems.

Wireless sensors have been considered for early flood alarming which are comprised of sensing elements, signal generators, and batteries.^[^ ^9^, ^10^, ^11^ ^]^ These sensors can respond promptly to rapidly developing floods and transmit the warning signals to the control center via wireless communication. Superior to cable supplies, the sensors powered by batteries are much cheaper and can be compatible with more complex occasions. However, the use of mobile batteries is also facing the issues of depletion during long‐term use and the short‐circuit caused by water damage, which severely invalidated the sensors for effective and early warning of floods. Therefore, a stable and long‐term energy supply is essentially needed to replace the batteries within the flood warning sensors. Renewable energy sources, such as wind and sunlight, have been once considered as sustained energy for flood warning sensors.^[^ ^12^, ^13^, ^14^ ^]^ However, these devices strongly rely on collecting external energy and limited by the large size, poor stability, and high cost, which are contrary to the requirement for actual applications in smaller or enclosed urban spaces such as bridges and tunnels. In this context, the early flood warning sensor is expected to require low or even no energy consumption at safe circumstances, while power itself in response to the flood occurrence, consistent with broad adaptability and long‐term guarantee.^[^ ^15^ ^]^ The way of harvesting the kinetic energy of floods and converting it as electrical energy was a competitive strategy of exploiting flood warning sensors with self‐powering ability. The sensors based on triboelectric or piezoelectric effect have been attractive for monitoring the change of water level, showing an agreement of self‐powering by flood energy.^[^ ^16^, ^17^, ^18^, ^19^, ^20^, ^21^, ^22^, ^23^, ^24^, ^25^, ^26^ ^]^ Allowing for the limited output of triboelectric and piezoelectric generators, it is challenging to activate more complex sensors with demands on higher energy consumption, particularly for continuous long‐distance signal transmission.

In this work, we developed an electrical sensor that could be switched from an asleep state to an awake state by water. The water‐triggered capability of the sensing system was attributed to a particular Galvanic battery which was electrically powerful only in the hydrated state. Referring to the principle of a Galvanic battery, a redox reaction accounting for the generation of voltage and current in the circuits occurs between an active metal and an inactive metal once they are bridged by an electrolyte solution. In other words, the battery stops working if the ionic conducting bridge between two electrodes is cut off. We herein prepared a paper‐based Galvanic battery made of a pair of copper‐zinc electrodes that was isolated by a copper sulfate‐loaded polyacrylamide (PAAM) xerogel. The battery could not generate electricity in dry conditions as the xerogel was an insulating layer for two electrodes, while it became a regular Galvanic battery if the xerogel was hydrated (Scheme 1c). Such a paper‐based battery could be folded into ones with smaller sizes and a higher number of battery units for easier portability and higher output power, respectively, following the craft of Miura origami. In the case of water levels rising over the warning level of the flooding at which the water‐triggered battery was placed, the battery was activated in a few seconds whose output power was high enough to trigger the sensing system consisting of signal generation and transmission modules.

## 2 Results and Discussion

### 2.1 Preparation and Characterization of a Water‐Triggered Xerogel from Insulative to Conductive

**Figure 1:**
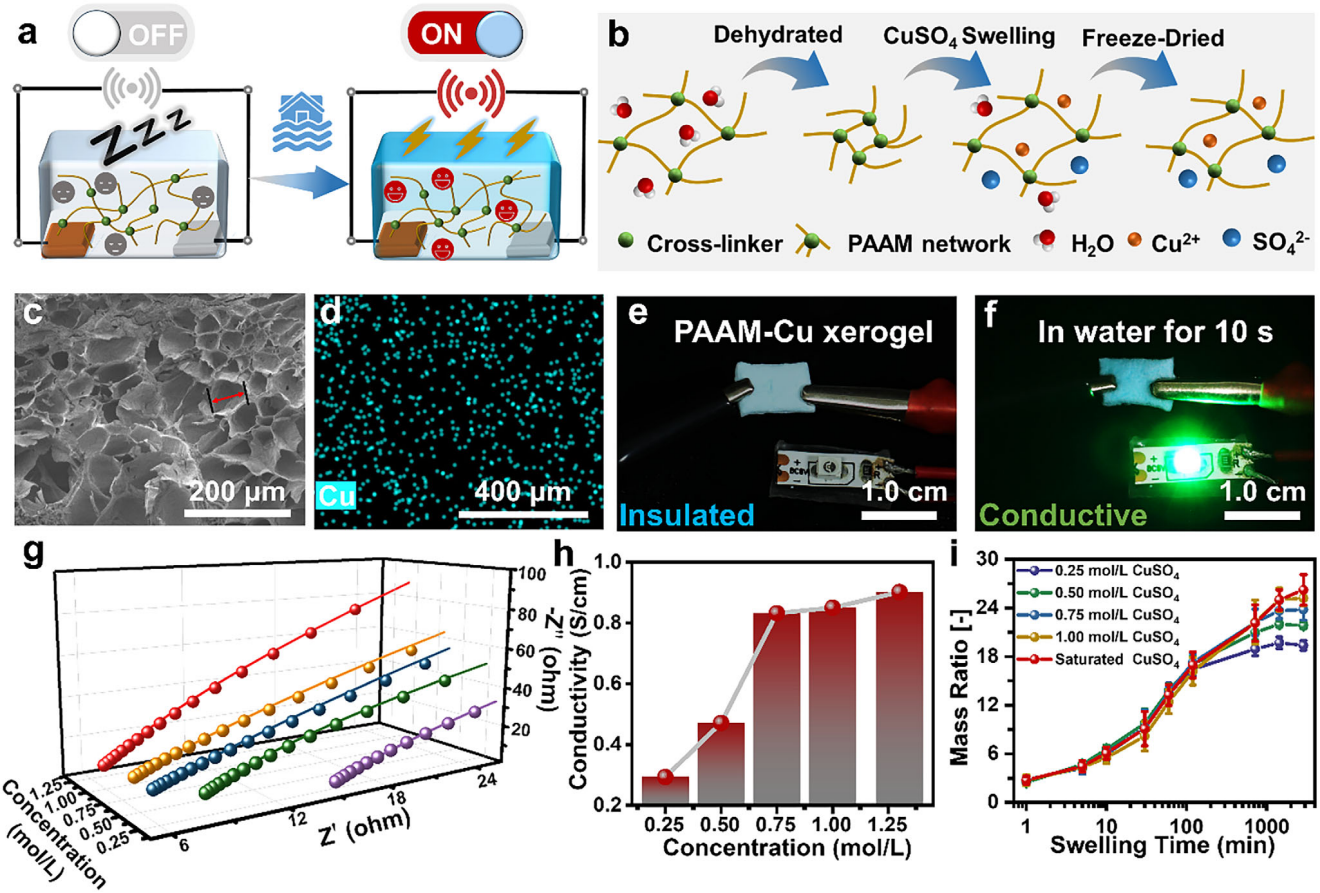
Preparation of a water‐triggered xerogel from insulative to conductive. a) Schematic of the principle of the self‐powered sensor based on hydrogel. b) The preparation process of PAAM‐Cu hydrogel. c) SEM image of PAAM‐Cu xerogel. The diameter of the core is ≈60 µm. d) EDS image of PAAM‐Cu hydrogel. e) The electrical circuit with a PAAM‐Cu xerogel and a Green LED. f) the electrical circuit with a PAAM‐Cu xerogel after immersion in water for 10 s and a Green LED. g) The electrical impedance spectroscopy of the PAAM‐Cu hydrogels with various concentrations. h) The conductivity of PAAM‐Cu hydrogels with various concentrations. i) Mass ratio of PAAM‐Cu hydrogels to PAAM‐Cu xerogels during swelling in different concentrations of copper sulfate solution.

The key part of the Galvanic battery for powering the flood alarming system is a copper sulfate‐loaded xerogel that isolates copper‐zinc electrodes. The xerogel is expected to have the capability of rapid water absorption to make an immediate response to floods (**Figure** **1**a). As illustrated in Figure 1b, the xerogel was a product of cross‐linked PAAM hydrogel after lyophilization, which was subsequently rehydrated by the saturated copper sulfate solution, followed by another lyophilization (see the preparation details in Figure S1, Supporting Information). Noting that Cu^2+^ can form coordination bonds with the cross‐linker, which ultimately disrupts the crosslinking of PAAM chains and prevents the formation of hydrogels (Figure S2a,b, Supporting Information).^[^ ^27^ ^]^ Furthermore, the effect of solution color on ultraviolet absorption was verified (Figure S2c,d Supporting Information). According to scanning electron microscopy (SEM) observations, the PAAM‐Cu xerogel has a porous structure with an average pore size of 60 µm as a result of water removal from the hydrogel (Figure 1c; Figure S3, Supporting Information). The successful loading of copper ions was verified by the energy dispersive spectrometer (EDS) spectrum in which the signals of copper element (light blue dots in Figure 1d) were well dispersed within the PAAM‐Cu xerogel, in contrast to the negligible copper signals within the PAAM xerogel (Figure S4, Supporting Information).

PAAM‐Cu xerogel exhibited different electrical properties of before and after immersion in water.^[^ ^28^ ^]^ As shown in Figure 1e, a PAAM‐Cu xerogel was bridged in a circuit monitored by a green LED. When the PAAM‐Cu xerogel was dry, the LED was off, corresponding to an open circuit. The extremely high resistance of the xerogel, measured with a multimeter, further confirmed its insulative property (Figure S5a, Supporting Information). When the xerogel was submerged in water for 10 s and then reconnected to the circuit, the green LED lit up. This was due to the decreased resistance of the xerogel when wet, as compared to its dry state (Figure 1f; Figure S5b, Supporting Information). The difference of the circuit from an insulative to a conductive state reflected the water‐triggered property of the PAAM‐Cu xerogel, which is attributed to its rapid and high water absorption capacity, owing to its porous structure (Figure S6, Supporting Information). Furthermore, electrochemical impedance spectroscopy tests were provided to investigate the impact of copper sulfate solution concentrations on the conductivity of PAAM‐Cu hydrogels, as shown in Figure 1g and Figure S7 (Supporting Information). By fitting the results to an equivalent circuit, it was found that the diffusion process dominated the bulk‐phase resistance, indicating that the conductivity of the hydrogel was closely related to the amount of copper sulfate loaded in the hydrogel (Figure S8, Supporting Information). Further analysis using Equation (1) revealed that the conductivity of the hydrogel decreases with higher concentrations of copper sulfate solution (Figure 1h).

(1) σ=L/SR

*σ* represents the conductivity of the hydrogel, *L* is the length of the hydrogel, *S* is the upper surface area of the hydrogel, and *R* is the resistance of the hydrogel. Additionally, the mass change of PAAM‐Cu xerogels as they swelled in copper sulfate solutions with varying concentrations was recorded in Figure 1i to examine the relationship between the amount of copper sulfate loaded into the hydrogel and the concentration of the copper sulfate solution. These results showed that the mass ratio of PAAM‐Cu hydrogels to PAAM xerogels also increased and stabilized over 24 h, as the concentration of copper sulfate solution increased. All of the above results demonstrate that copper sulfate can be effectively incorporated into the PAAM‐Cu gel framework with increasing concentration, corresponding to an increase in hydrogel conductivity and enabling the long‐term and rapid activation of the water‐triggered batteries.^[^ ^29^ ^]^

### 2.2 Preparation of the Water‐Triggered Galvanic Battery on Paper

**Figure 2:**
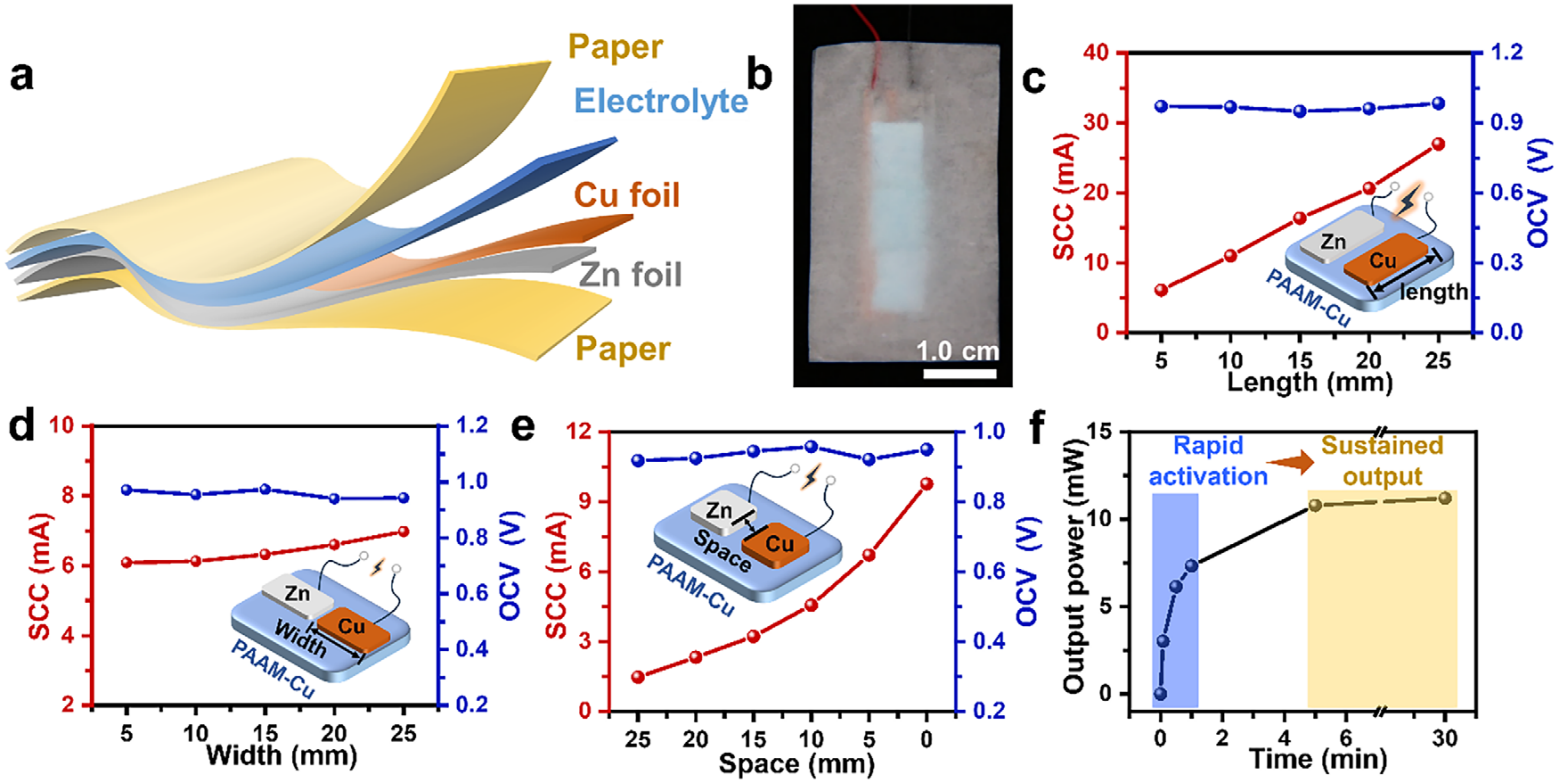
Preparation and electrical optimization of the water‐triggered Galvanic battery on paper. a) Multi‐layered structural design of battery unit. b) Picture of a battery unit. The length, width, and height of both of copper and zinc foils are 5.0, 10.0, and 1.0 mm, respectively, and that of the PAAM‐Cu xerogels are 5.0, 5.0, and 2.0 mm, respectively. c‐e) Influence of varying metal electrode lengths (c), widths (d) the spaces between electrodes (e) on the short‐circuit current (SCC, left) and open‐circuit voltage (OCV, right) of battery. f) Output Power of battery unit (The length, width, and height of both of copper and zinc foils are 5.0, 30.0, and 1.0 mm, respectively, and that of the PAAM‐Cu xerogels are 5.0, 5.0, and 2.0 mm) for different times of dissolution in water.

As shown in **Figure** **2**a, the water‐triggered Galvanic battery consists of a zinc foil, a layer of PAAM‐Cu xerogel, and a copper foil placed on a paper base. Redox reactions at the two metal/xerogel interfaces are activated once the xerogel is hydrated, corresponding to an “awakened” galvanic battery, in which the zinc foil and copper foil function as the cathode and anode, respectively.^[^ ^30^, ^31^ ^]^ The battery performance is closely related to physical parameters such as the length (parallel to the other electrode, as shown in the inset of Figure 2b), width (perpendicular direction), and thickness of the electrodes (Figure S9, Supporting Information). The results demonstrated that the short‐circuit current of the battery units increased linearly from 6.1 to 26.9 mA as the electrode foil length was extended, whereas negligible changes were observed with variations in electrode foil width and thickness. Additionally, no significant impact was observed on the open‐circuit voltage of the battery units with different electrode sizes (Figure 2c,d; Figure S10, Supporting Information). These findings can be explained by the fact that a longer foil length increases the number of parallel circuits in the equivalent circuit of the battery units (Figure S11, Supporting Information). The voltage of the units primarily depends on the oxidation‐reduction reactions occurring within the battery. These results highlight an effective structural design for Galvanic batteries to enhance electrical output, a factor that has been rarely addressed in previous research.^[^ ^32^ ^]^ Furthermore, the voltage remained almost unchanged when the distance between the electrode foils were reduced, whereas the current increased significantly, reaching 9.7 mA under the condition of minimal spacing (Figure 2e). This can be explained by Equation (2), when the distance between the electrode foils is smaller, the ions face less resistance from the gel network, leading to faster migration within the gel and consequently a higher current.

(2) I=nSqv

*n* is the number of free charges per unit volume, *S* represents the cross‐sectional area of the conductor, *q* is the average charge per free ions, *v* is the velocity of a free charge in a conductor. Ultimately, other impacts also were explored to optimize the output of the battery. A higher current was observed for the PAAM xerogel treated with a higher concentration of copper sulfate solution while the voltage was almost retained unchanged (Figure S12, Supporting Information). Such an increased current upon loading more copper sulfate within the xerogel can be explained by Equation (2), in which *n* is increased. Based on these experiments, the optimal structure for the sensor units was specified (Figure 2b). When the water‐triggered battery was immersed in water, it generated a remarkable power output of 3.0 mW within 5 s, reaching 10.8 mW within 5 min. Moreover, it maintained a stable power generation of 11.2 mW after 30 min, meeting the requirements for both rapid and long‐term flood warnings (Figure 2e).

### 2.3 Enabling the Water‐Triggered Battery Humidity Protection

**Figure 3:**
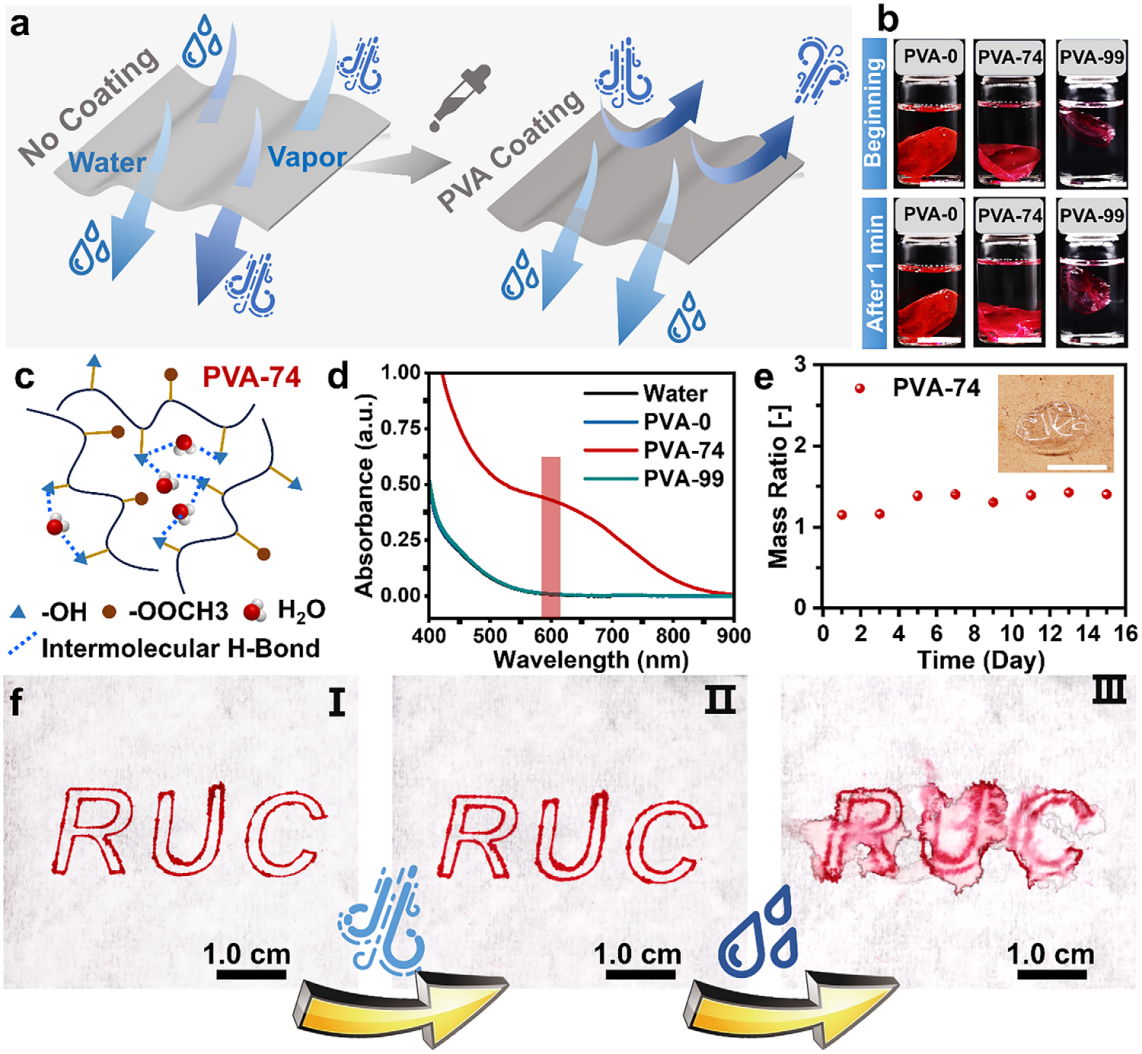
Material design that combines anti‐humidity and water permeability for the water‐triggered battery. a) Schematic of ideal coating prevents the passage of moisture (left) but can be dissolved in water (right). b) Dissolution of the films of PVA‐0, PVA‐74, and PVA‐99 after dissolving in water for 1 min after dyeing with Nile Red. Scale bar: 2.0 cm. c) Mechanism of PVA‐74 film dissolves in water. d) UV–vis spectra of water and the films of PVA‐0, PVA‐74, and PVA‐99 dissolved in water after 1 min. e) Mass ratio of PVA‐74 films after continuous treatment at high humidity environment (RH = 90%) compared with untreated samples. Inset: the picture of PVA‐74 film after continuous treatment at a high humidity environment (RH = 90%). Scale bar: 2.0 cm. f) Anti‐humidity and permeable performance test: I) PVA‐74 was applied on one side of the filter paper with “RUC” written on the on opposite side; II) this filter paper was placed in a sealed box with high humidity environment (RH = 90%); III) water was applied to the PVA‐74 coated side of the filter paper.

Floods are often caused by persistent and heavy rainfall, which indirectly leads to a significant and sustained increase in humidity. The high humidity may induce a false triggering of xerogels and electrical sensors, while also corroding metal electrodes, thereby compromising the reliability of early flood warning systems. Therefore, a material that rapidly dissolves in water for timely activation while maintaining resistance to humidity is required for the sensing system. Prior research has shown that polyvinyl alcohol (PVA) exhibits humidity insensitivity by forming a gel‐like polymer polarization layer at the interface (**Figure** **3**a).^[^ ^33^, ^34^, ^35^, ^36^ ^]^

The different alcoholysis degrees of PVA are derived from the alcoholysis reaction of polyvinyl acetate (PVAc), whose water solubility was tested as follows. The product with a 3:1 ratio of hydroxyl groups to vinyl acetate units was designated as PVA‐74. The sample with nearly complete alcoholysis was named PVA‐99, and PVAc was referred to as PVA‐0 for convenience and the NMR spectra of them were displayed in Figure S13 (Supporting Information). As shown in Figure 3b, three solid PVA films, after being dyed with Nile Red and fully dried, were immersed in water for 60 s. A distinct color change was observed in the aqueous solution of the PVA‐74 film compared to PVA‐0 and PVA‐99 films, indicating that only the PVA‐74 film exhibits favorable water solubility. Additionally, the PVA‐74 film was fully dissolved after immersion in water for 24 h (Figure S14, Supporting Information). This is due to the large number of hydrophobic vinyl acetate groups in the PVA‐0 film, which weakens its affinity with water. In contrast, the strong intramolecular hydrogen bonds in the PVA‐99 film make it difficult to dissolve in water (Figure S15, Supporting Information). On the other hand, the optimal number of hydroxyl groups in the PVA‐74 film allows for the formation of stable intermolecular hydrogen bonds with water molecules, rather than intramolecular hydrogen bonds, thereby enhancing its water solubility and promoting easier dissolution in water (Figure 3c). To further identify the water solubility of these PVA films, a UV–vis spectrum was performed on the extraction solution treated with chromogens of the unstained PVA films dissolved in water for 60 s (Figure 3d; Figure S16, Supporting Information).^[^ ^37^, ^38^ ^]^ In Figure 3d, a distinct absorption peak ≈600 nm is observed for the PVA‐74 film (red region), which corresponds to a specific absorption feature arising from the complex formed between PVA and the chromogens. Further analysis, after immersing these films in water for 24 h, also confirmed this result (Figure S17, Supporting Information). These findings collectively demonstrate the excellent water solubility of the PVA‐74 film and indicate that it does not interfere with the activation of the water‐triggered battery.

The mass change of PVA‐74 films was continuously monitored over 15 days at a relative humidity (RH) of 90% to evaluate their anti‐humidity properties. As shown in Figure 3e, the PVA films did not exhibit deliquescence or shape distortion, and the mass change of the PVA films increased slightly. These results suggest that the PVA‐74 films provide anti‐humidity protection for both the metal electrode and the xerogels in the water‐triggered battery, ensuring reliable performance in high‐humidity environments without the risk of degradation. Furthermore, the anti‐humidity and water solubility of PVA‐74 films were displayed intuitively. As shown in Figure 3f, filter paper was partially covered with PVA‐74 solution and fully dried to obtain one side of the paper coated with PVA‐74. Then, the letters “RUC” were written on the other side using a rollerball pen (Figure 3f‐I). The PVA‐74 coated side was subsequently exposed to an RH = 90% environment for 7 days, while the untreated side remained sealed. After 7 days, the “RUC” writing remained clear on the untreated side (Figure 3f‐II). When water was introduced to the PVA‐74 coated side, the written “RUC” became significantly blurred within seconds (Figure 3f‐III). This observation further confirms the superior anti‐humidity and water solubility of PVA‐74, demonstrating its ability to meet the demand of normal operation of the battery in various environments.

### 2.4 Integrated Water‐Triggered Batteries with High Power Output

**Figure 4:**
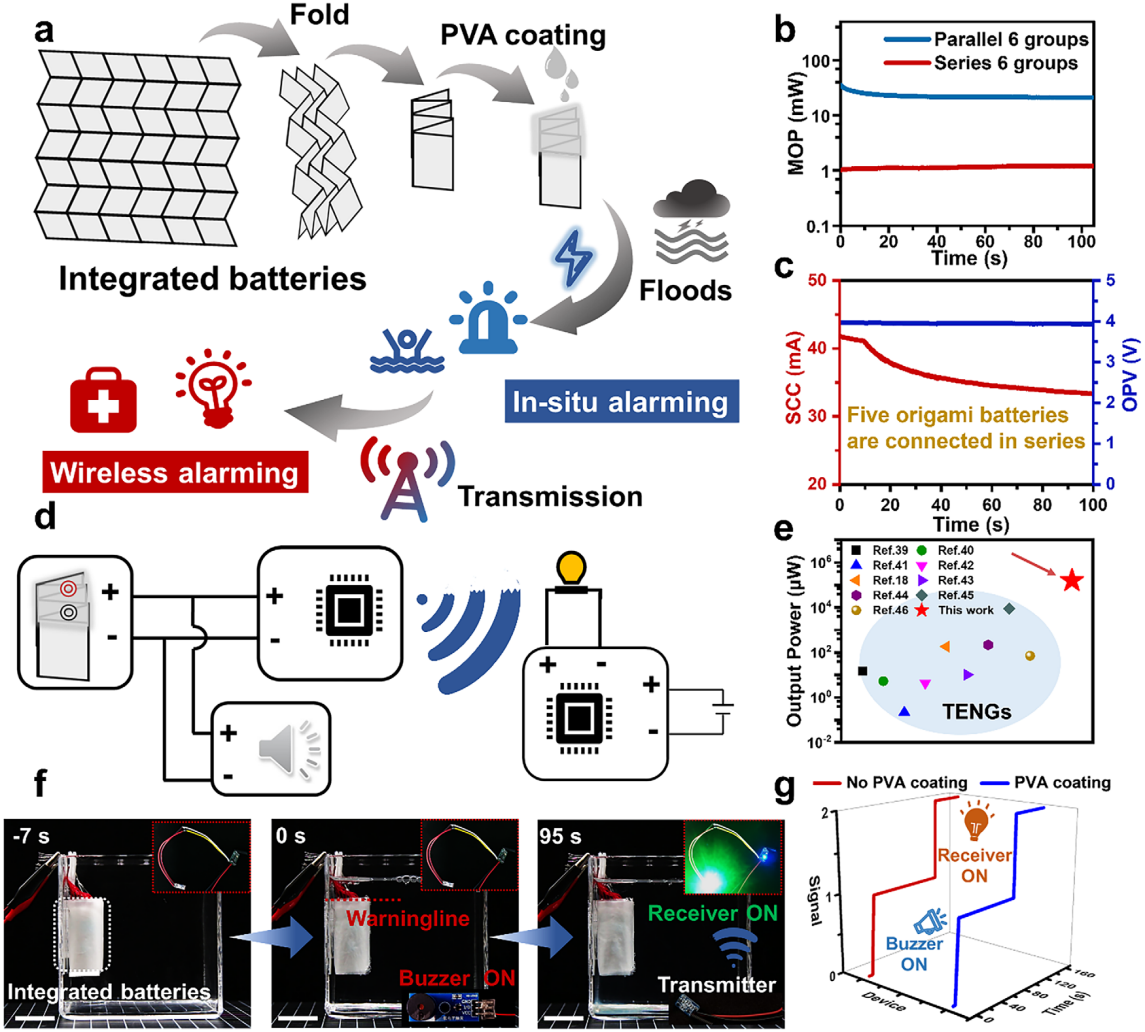
The integration of water‐triggered batteries and its indoor experiment testing. a) The integration of the water‐triggered battery inspired by Miura origami with dual flood warnings of buzzer and wireless transmission. b) Comparison of the output power of water‐triggered batteries in series and parallel. c) Current (left) and voltage (right) output of integrated water‐triggered batteries. d) Equivalent circuit of the self‐powered flood warning system. e) The output power comparison with other self‐powered flood warning sensors. f) Indoor experimental demonstration of the integrated water‐triggered batteries. Scale bar: 2.0 cm. g) Comparison of the response time of the integrated water‐triggered batteries with PVA‐74 film coating and without PVA‐74 film coating.

The design of water‐triggered batteries, as described above, satisfies the reliability requirements in high‐humidity environments and enables rapid electrical response upon hydration. In real‐world urban flood scenarios, dual flood alerts, including an in situ audible alarm for pedestrians and a wireless alarm for communication with a remote control center, are critically important and necessary (**Figure** **4**a). Consequently, flood warning sensors typically require high power input, miniaturization, and high integration to ensure their practical applicability. Natural insects commonly fold their large wings into their carapaces, featuring a small folded area, which can be rapidly expanded into a large unfolded area. Inspired by this mechanism, we utilized Miura origami for the integrated array design of water‐triggered batteries. This approach effectively minimized the occupied area of water‐triggered batteries while simultaneously achieving their high output power for dual alerts.

The dual warning devices are primarily composed of a buzzer for audible alarm, a wireless signal transmitter, and a signal receiver for sending signals to the remote control center (Figure S18, Supporting Information). Subsequently, the electrical integration of water‐triggered batteries was implemented in two configurations: either six water‐triggered batteries connected in series or in parallel. The electrical results showed no significant difference in open‐circuit voltage between the two configurations. However, the short‐circuit current of the parallel circuit was more than twenty‐two times higher than that of the series circuit (Figure S19, Supporting Information). This difference was explained by the fact that water itself was a conductor, leading to the partial short‐circuiting in batteries connected in series. This is why the parallel configuration in the Miura origami arrangement of batteries is distinctly advantageous (Figure 4b). The straightforward fabrication process for the water‐triggered batteries is detailed in Figure S20 (Supporting Information). Considering that the requirement of high voltage input for dual alerts (Figure S21, Supporting Information), a secondary series integration of five water‐triggered batteries was implemented, resulting in a voltage of up to ≈4 V and a current exceeding 40 mA, thereby fully meeting the power requirements of the flood warning system (Figure 4c). The maximum power of the integrated water‐triggered batteries was also determined by connecting an external load. By varying the resistance of the external load, the voltage and current from the integrated batteries were measured, revealing that the maximum output power density was ≈2.54 W m^−2^ (Figure S22, Supporting Information). The buzzer and the signal transmitter were designed in parallel into the circuit, enabling these highly integrated batteries to quickly achieve double warnings (Figure 4d). Notably, the output power of the integrated water‐triggered batteries was significantly higher than that of other reported self‐powered flood warning devices, underscoring the unique advantage of the water‐triggered battery (Figure 4e).^[^ ^18^, ^39^, ^40^, ^41^, ^42^, ^43^, ^44^, ^45^, ^46^ ^]^

Additionally, the integrated water‐triggered batteries, subjected to anti‐humidity treatment, were tested to verify their feasibility in a simulated flood scenario. When the water level reached the designated water level threshold, the buzzer was rapidly activated within 7 s, while wireless signal transmission began 95 s after the water level reached the warning point (Figure 4f; Movie S1, Supporting Information). The activation of the integrated batteries within two minutes demonstrated their reliability of them for dual alerts. Furthermore, the integrated batteries without anti‐humidity treatment were tested under identical conditions to assess the impact of the anti‐humidity design of the PVA‐74 film on the timeliness of activation. The response time of the non‐treated batteries was nearly identical, with only a 2 s difference in response time. These results suggested that the PVA‐74 film had a negligible effect on the activation performance of the water‐triggered battery (Figure 4g).

### 2.5 The Self‐Powered Flood Warning Sensor in the Outdoor Flood Scenario

**Figure 5:**
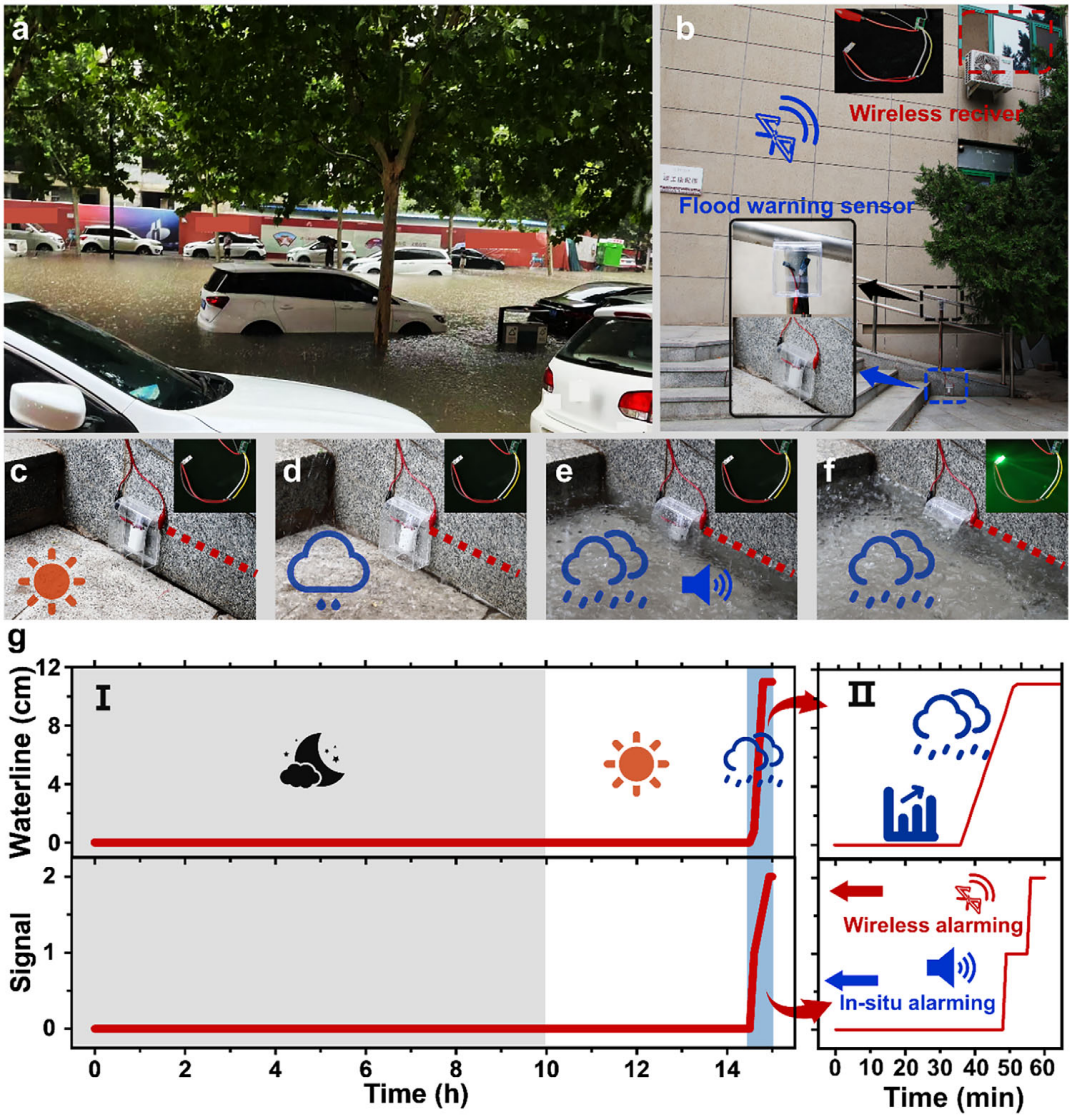
Performance of the self‐powered flood warning sensor in the outdoor flood scenario. a) Photo of the case when urban flooding occurs. The picture was captured in Kaifeng, China. b) Simulated scenario of urban flooding and self‐powered flood warning sensor setup. c) The self‐powered flood warning sensor on a sunny day. d) The self‐powered flood warning sensor when raining. e) The self‐powered flood warning sensor when the rain increases and the water level reaches the warning line, the buzzer is corresponding. f) Response of the signal receiving system at the far end when the water level floods the warning line. g) Water level as well as signal changes throughout the process. I) Water level and signal changes in different weather conditions. II) Water level and signal changes in the last hour.

Urban floods are both devastating and unpredictable, primarily due to the concentration of population in urban areas and the complex topography of these environments (**Figure** **5**a). As a result, the flood warning sensor must be reliable and accurate in various environmental conditions. Large‐scale rainfall often leads to the formation of unpredictable deep water in low‐lying urban areas, which causes severe urban floods. To address this risk, a waterproof cover was utilized in conjunction with integrated water‐triggered batteries, effectively preventing false warnings caused by raindrops (Figure S23, Supporting Information).

To further validate the feasibility of a self‐powered flood warning sensor in a real flood environment, a flood scenario was simulated using artificial rainfall, and the integrated water‐triggered batteries along with the alarming devices were pre‐installed (Figure 5b). As illustrated in Figure 5c,d, the self‐powered flood warning sensor gave no false alarm on sunny days and remained unresponsive during light rain. When long‐term rainfall resulted in flooding and the water level reached the critical threshold, the buzzer was quickly activated to warn people to stay away (Figure 5e; Movie S2, Supporting Information). Shortly afterward, the wireless signal was transmitted to the remote receiver. This rapid activation of both the buzzer and the wireless signal effectively protected people from urban flooding and ensured timely flood prevention measures, significantly reducing flood‐related losses (Figure 5f). This effectiveness is further demonstrated by the quantitative data from long‐term monitoring. The rainfall and signal output were recorded under nighttime, sunny, and rainy conditions. No output signal was detected during nighttime or sunny days, as shown in Figure 5g‐I. However, when the water level surpassed the warning threshold, both the buzzer and signal transmitter responded rapidly, as illustrated in Figure 5g‐II. Additionally, changes in ambient noise levels were recorded, and an increase of 10 dB was triggered by the buzzer (Figure S24, Supporting Information). This corresponded to a threefold increase in the perceived loudness of the sound, effectively warning pedestrians about potential flooding outdoors. Overall, by simulating urban flooding caused by heavy rainfall, the reliability of the self‐powered flood warning sensor under normal conditions and its ability to provide rapid dual warnings during a flood event were validated. To assess the long‐term stability of the sensor, the electrical tests of the flood warning sensor were performed outdoors. As shown in Figure S25 (Supporting Information), no significant electrical signals were detected from the sensor for two weeks and the output power of ≈180 mW was obtained by adding water to the sensor on the sixteenth day, indicating the sensor exhibits long‐term operational stability. Furthermore, the long‐term stability of the sensor under RH = 90% environment was also confirmed in Figure S26 (Supporting Information). This demonstrates that the self‐powered flood warning sensor holds significant promise for future urban flood early‐warning systems.

## 3 Conclusion

In summary, we have developed a low‐cost, self‐powered flood warning sensor that innovatively integrated a water‐triggered battery with dual alarming devices. The water‐triggered battery based on the principle of a Galvanic battery could maintain no energy consumption in dry conditions and only generate high output power when floods coming, overcoming the application limitations of traditional flood warning systems. By employing a straightforward polymerization and lyophilization process, a scalable and cost‐effective production of battery components were achieved, addressing the complexity and high costs associated with conventional flood warning sensors. The introduction of PVA‐74 films provided anti‐humidity protection for both the metal electrodes and xerogels in the water‐triggered battery, ensuring reliable performance in high‐humidity environments without degradation. Additionally, the integration of Miura origami in the water‐triggered battery design enabled significant space minimization while enhancing output power, thereby broadening the potential range of applications. This sensor showed great promise as a sustainable power source for the development of intelligent urban flood warning networks. Furthermore, all components of the water‐triggered battery, including the paper‐based substrate, hydrogels, and copper sulfate, exhibit outstanding environmental compatibility, aligning with societal demands for low‐pollution and eco‐friendly electronic products. We are confident that this advancement substantially enhances our ability to combat floods, contributing to the protection and well‐being of communities worldwide.

## Conflict of Interest

The authors declare no conflict of interest.

## Supporting information



Supporting Information

Supplemental Movie 1

Supplemental Movie 2

## Data Availability

The data that support the findings of this study are available in the supplementary material of this article.
