# Supplementary material for: A Water‐Triggered Sensor for Self‐Powered Flood Alarming
Source: Adv Sci (Weinh). 2025 Jul 9;12(30):e03815. doi: 10.1002/advs.202503815 (PMC12376692; doi:10.1002/advs.202503815)
Supplement: Supplementary file 1 — Supporting Information [file ADVS-12-e03815-s003.docx]

A water-triggered sensor for self-powered flood alarming

Mingqi Zhao, Shanzhi Lyu*, Yingchao Ma, Nan Zhang, Yapei Wang*

M. Zhao, S. Lyu, Y. Ma, N. Zhang, Prof. Y. Wang

School of Chemistry and Life Resources, Renmin University of China, 100872 Beijing, China
E-mail: [yapeiwang@ruc.edu.cn](mailto:yapeiwang@ruc.edu.cn)

S. Lyu

Department of Energy and Power Engineering, Tsinghua University, 100082 Beijing, China.

**1.Experimental Section**

**1.1 Preparation process of PAAM-Cu xerogel**

First, 2.0 g of acrylamide (Energy Chemical, W610515), 20.0 mg of Irgacure 2959, 5.0 mg of N, N'-methylene bis(acrylamide) (MBA), and 10.0 mL of deionized water were sequentially added to a vial. The mixture was then sealed under a nitrogen atmosphere and subjected to UV light irradiation for 60 minutes to initiate polymerization. Following the polymerization, the resulting polyacrylamide (PAAM) hydrogel was thoroughly dried in an oven at 60°C for 2 hours.

Subsequently, the dried PAAM hydrogel was immersed in a saturated copper sulphate solution (prepared by dissolving 20.0 g of anhydrous copper sulphate (Xi Long Scientific, 500.0 g) in 100.0 mL of deionized water) for 24 hours to allow for complete swelling, thereby forming the PAAM-Cu hydrogel. After swelling, the PAAM-Cu hydrogel was cut into approximately 0.5 cm squares using a ruler. The squares were then frozen in a freezer before being transferred to a freeze-dryer, where they were lyophilized for 12 hours. Finally, the PAAM-Cu xerogels were successfully obtained.

**1.2 Characterizations of the PAAM-Cu xerogel**

The microstructure and elemental composition of the PAAM and PAAM-Cu hydrogels were analyzed using scanning electron microscopy (SEM, SU8010, Hitachi), equipped with energy-dispersive X-ray spectroscopy (EDS, XFlash 6160, Bruker). The ultraviolet (UV) absorption spectra of PVA samples with varying degrees of alcoholysis, dissolved in water, were recorded using a SHIMADZU UV-3600 spectrometer. In this procedure, 1.0 mL of the dissolved PVA solution was mixed with 3.0 mL of a 0.6 mol/L boric acid solution, followed by the addition of 1.0 mL of a 0.01 mol/L I/KI solution to initiate the color development reaction. Finally, 5.0 mL of water was added to complete the mixture. The mass change of the PAAM and PAAM-Cu xerogels during swelling in water was monitored using an electronic balance (AH-A+R, China).

The swelling process of the PAAM xerogels in different concentrations of copper sulphate solution would be provided. First, the PAAM hydrogel obtained through UV photopolymerization was fully dried in an oven to produce PAAM xerogels. These xerogels were then immersed in copper sulfate solutions of varying concentrations to undergo the swelling process. At designated time intervals, the xerogels were removed and weighed to determine their mass after swelling. This allowed us to calculate the swelling mass ratio at different time points.

**1.3 Electrical optimization**

The voltage and current outputs of the water-triggered battery were measured using a CHI660e electrochemical workstation (Shanghai CH Instrument Co., Ltd.). In the evaluation of the electrode sheet length within a single sensing unit, the width of the clipped electrode sheet was fixed at 0.5 cm, while the lengths were varied from 0.5 cm to 2.5 cm in 0.5 cm increments. Voltage and current outputs were recorded for each electrode length. For the investigation of electrode sheet width, the length was held constant at 0.5 cm, and the thickness was maintained at 0.1 mm, while the width was incrementally increased from 0.5 cm to 2.5 cm in 0.5 cm intervals. The corresponding voltage and current outputs were then measured. To optimize electrode spacing, the distance between two electrode sets was adjusted by controlling the electrode length at 1.0 cm, the width at 0.5 cm, and the thickness at 0.1 mm. The spacing was varied from 0.5 cm to 2.5 cm in 0.5 cm increments, with voltage and current recorded at each interval. Subsequently, the electrode length was maintained at 1.0 cm and the width at 0.5 cm, while the thickness was varied from 0.1 mm to 0.5 mm in 0.1 mm increments. The voltage and current outputs were measured for each electrode thickness.

In all experiments, the PAAM hydrogels were fully swollen in copper sulphate solution. The copper sulphate concentration was varied, with the PAAM hydrogel swollen in solutions of 0.1 mol/L, 0.25 mol/L, 0.5 mol/L, 0.75 mol/L, and 1.0 mol/L copper sulphate. After complete swelling, the dimensions of the PAAM hydrogel were 1.0 cm in length and 0.5 cm in width. Electrical tests were conducted on electrodes with a thickness of 0.1 mm. During the testing, the temperature was maintained at 21.7°C, and the relative humidity was kept constant at 11%.

**1.4 PVA film dissolution experiment**

PVAc (polyvinyl acetate) (MACKLIN, P823279-100g), also named as PVA-0, is the precursor of PVA. PVA-74 (Aladdin, P139547-500g) refers to the PVA-0 with an alcoholysis degree between 74% and 80%, and is named PVA-74 for convenience. PVA-99 (Shanghai Titan chem Co., Ltd. G88945) refers to the PVA-0 with an alcoholysis degree of 99%. 2.0 g of PVA-0 solid was taken and 20.0 g chloroform was added to the glass bottle, which was stirred at 60 ℃ with a magnetothermal mixer for 3 h until it was fully dissolved. 2.0 g of PVA-74 solid was taken respectively, and 20.0 mL water was added to the glass bottle, which was stirred at room temperature with a magnetothermal mixer. Then 2.0 g of PVA-99 solid was taken respectively, and 20.0 mL water was added to the glass bottle, which was stirred at 90℃ with a magnetothermal mixer for 3 h until it was fully dissolved.

About 1.0 g of the above mixed solution was taken and dropped into the glass bottle and was put into a blast oven for continuous treatment at 60 ℃ for 3 h. Moreover, 15.0 mL of pure water was added into the glass bottle, and the dissolution process was recorded. 4.0 mL of the extraction solution was removed after 1min of dissolution, 3.0 mL of boric acid solution (0.6 mol/L), 1.0 mL of KI/I_2_ (0.01 mol/L) and 12.0 mL of water was added in a glass bottle for UV-VIS. 1.0 mL of the extraction solution was removed, 5.0 mL of boric acid solution (0.6 mol/L), 1.0 mL of KI/I_2_ (0.01 mol/L) and 12.0 mL of water were added in a glass bottle for UV-VIS after 24 h of dissolution. In order to visually observe the dissolution process of film, Nile red (HEOWNS, N38400MGCI) was used as a probing dye. During the dissolution process of PVA with different alcoholysis degrees, about 2.0 mg of Nile red was added to each group. After full dissolution, the film was dried in the oven according to the above steps, and the dissolution process was recorded. All physical images were recorded by a camera (Canon DS12601).

**1.5 The preparation of origami batteries**

Firstly, a hydrophobic paper is used as the base of the sensor. The length of the oil absorbing paper is about 7.5 cm × 20.0 cm, and then folded into a 3 × 2 rectangular square according to Miura origami. We used liquid glue (purchased from De li) to fix the high purity copper and zinc sheets to the base. The size of copper and zinc sheets (purchased from Kuo tang Metal Materials Trading Company, Yao hai District, Hefei City) was cut to 0.5 cm × 3.0 cm × 0.1 mm, and the spacing between copper and zinc sheets is about 0.1 cm, but they were not in contact. We then cut the wire to a length of about 3.0 cm, using the welding gun and solder wire to weld the wire to each metal sheet. PAAM-Cu xerogel prepared above was fixed with copper-zinc sheet using a small amount of liquid glue. Next, origami battery units were folded and grew to about 5.0 cm, with a width of about 2.5 cm and a thickness of about 1.5 cm, and five origami batteries were welded in series to finally obtain the integrated batteries.

**1.6 Humidity control experiment**

Humidity Regulation: An air humidifier, coupled with a humidity monitor (C02), was situated inside a large, sealed plastic container. This setup allowed for precise control of the humidity levels. The humidifier was activated when the humidity fell below the predetermined set point and ceases operation once the humidity exceeded this value. Utilizing this system, the hygroscopic properties of the PAAM-Cu xerogel and PVA-74 thin film were investigated at a targeted humidity of approximately 90%. Concurrently, in an experiment to study the deliquescence and hydrolysis effects of PVA-74 using filter paper, the side treated with PVA-74 was exposed to an environment with a humidity of 90%. To isolate the effect, the opposite side, which featured RUC markings made with a red carbon water-based pen, was partially sealed with nano-tape in conjunction with a plastic cup. This arrangement ensured that water vapor could only penetrate the substrate from the PVA-74 coated side.

**1.7 Laboratory experiment**

An indoor experiment was designed to mimic the scenario of flooding during extreme weather events by rapidly introducing water into an array of units within a transparent glass tank, thereby inducing a change in water level. The integrated batteries were treated by PVA-74 for the anti-humidity. The PVA-74 solution was prepared through mixing with water in a ratio of 1:5 and the upper of the integrated batteries was treated with the PVA-74 solution, followed by the formation of a PVA film after drying in the oven. The integrated batteries were secured to the side wall of a clear glass cylinder (10.0 cm in edge length) with nano tape (M&g, AJD957F6). The array was positioned at a height of approximately 6.0 cm, and it was connected to a buzzer (Shenzhen Beike Trading Co., Ltd) and a signal transmitter (Shenzhen You Kong Electronic Technology Co., Ltd). In a separate setup, a remote signal receiving device was installed, which comprised a signal receiver and a green LED indicator. As water was added to the glass cylinder, once the water level reached the predetermined alarm threshold, the buzzer responded promptly with an alarm within 7 s. Following a brief interval of 95 s, the signal transmitter dispatched the alert, and the signal receiver picked up this transmission, illuminating the LED as a result. The temperature during the experiment was 25 ℃ and the humidity was 62%.

**1.8 The water-triggered battery with PVA coating**

It has been proved that PVA-74 film had a great potential in moisture resistance, so we applied PVA-74 coating treatment to the water-triggered batteries which had been integrated. We configured a PVA-74 aqueous solution with a sample-to-water mass ratio of 1:5. Since PVA-74 aqueous solution with a higher mass ratio had a higher viscosity, we used a dropper to apply a layer of PVA-74 film to the top of the water-triggered batteries, and then put it into an oven to dry before further uses.

**1.9 Outdoor Experiment**

In the outdoor experiment, the test site was selected at the entrance of the auxiliary building of the Science and Engineering Building at Renmin University of China. The integrated water-triggered batteries were connected to a buzzer and a signal transmission device, while the signal receiving device was positioned inside a second-floor room. We built a sealed space with a volume of about 84 L using bricks and a small amount of discarded plastic bags, and then used a water gun to simulate the rain process. The simulated rain rate of the water gun was 4 L/min, and the process of water accumulation in low-lying roads was simulated by the external backflow of rainwater from high places. The integrated water-triggered batteries exhibited no false alarms during both clear daytime and nighttime conditions. After being exposed outdoors for over 12 h, a simulated artificial rainfall was introduced. Approximately 12 min into the rain, the water level reached the alert threshold, triggering an immediate response from the buzzer, which resulted in a sound intensity increase of about 10 dB. Then, after an additional 8 min, the signal receiver picked up the transmission. This delay was attributed to the increased power required to initiate the signal transmitter due to the transmission distance. This aligns with the observed increase in power output from the water-trigged battery units as the swelling time progresses within a certain timeframe. The temperature during the experiment was 29 ℃ and the humidity was 58%.


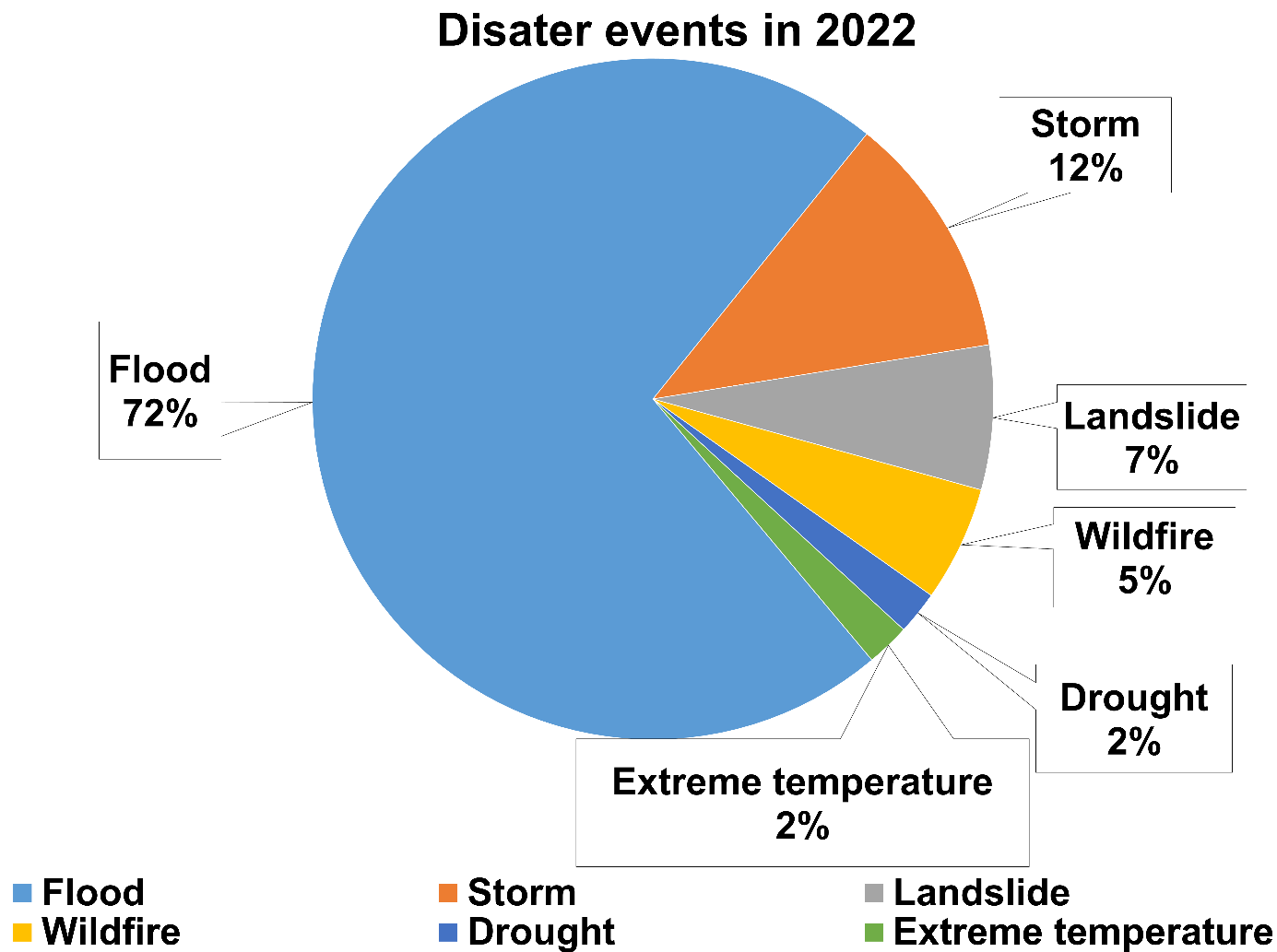
**、**

**Table S1** Statistical map of the number of natural disasters occurring globally in 2022.


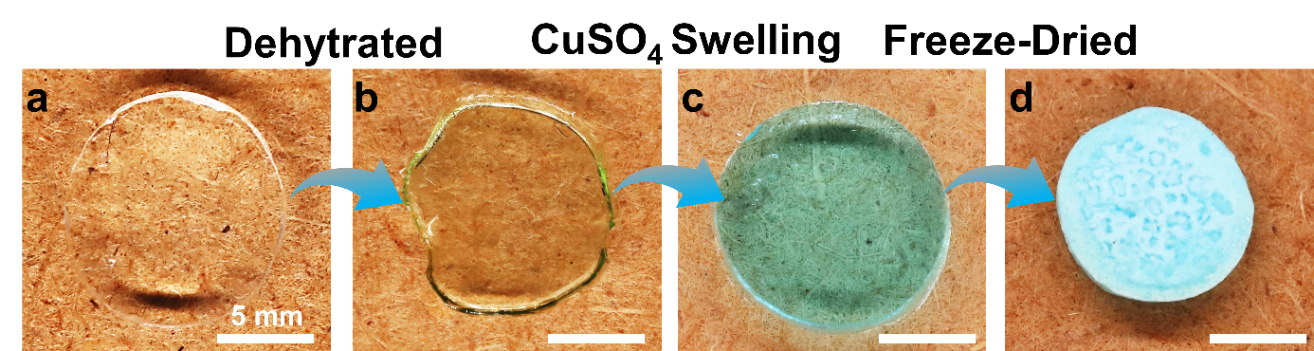


**Figure S1.** Preparation of PAAM-Cu gels. a) PAAM hydrogel obtained by photo-polymerization for 1 hour under ultraviolet light at a power of 28 mW. b) PAAM xerogel dried for 3 h in a blower oven. c) PAAM-Cu hydrogel immersed in saturated CuSO₄ solution for 24 h. d) PAAM-Cu xerogel freeze-dried for 12 h. Scale bar: 5.0 mm.


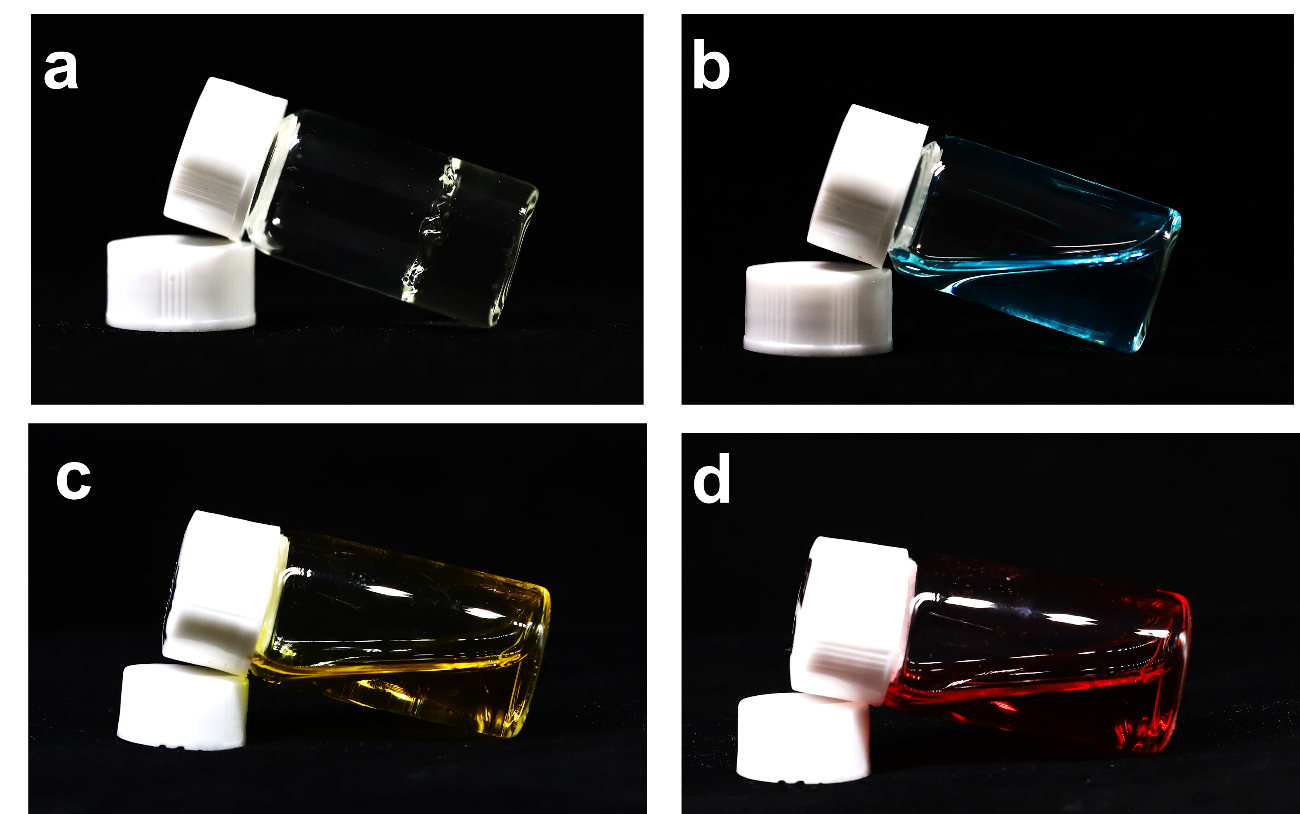


**Figure S2.** a) PAAM hydrogel obtained by photo-polymerization without copper sulphate. b) Product obtained through photo-polymerization of PAAM prepolymer solution mixed with solid copper sulphate at a concentration of 0.5 mol/L. c) Product obtained through photo-polymerization of PAAM prepolymer solution mixed with tartrazine. d) Product obtained through photo-polymerization of PAAM prepolymer solution mixed with carmine.


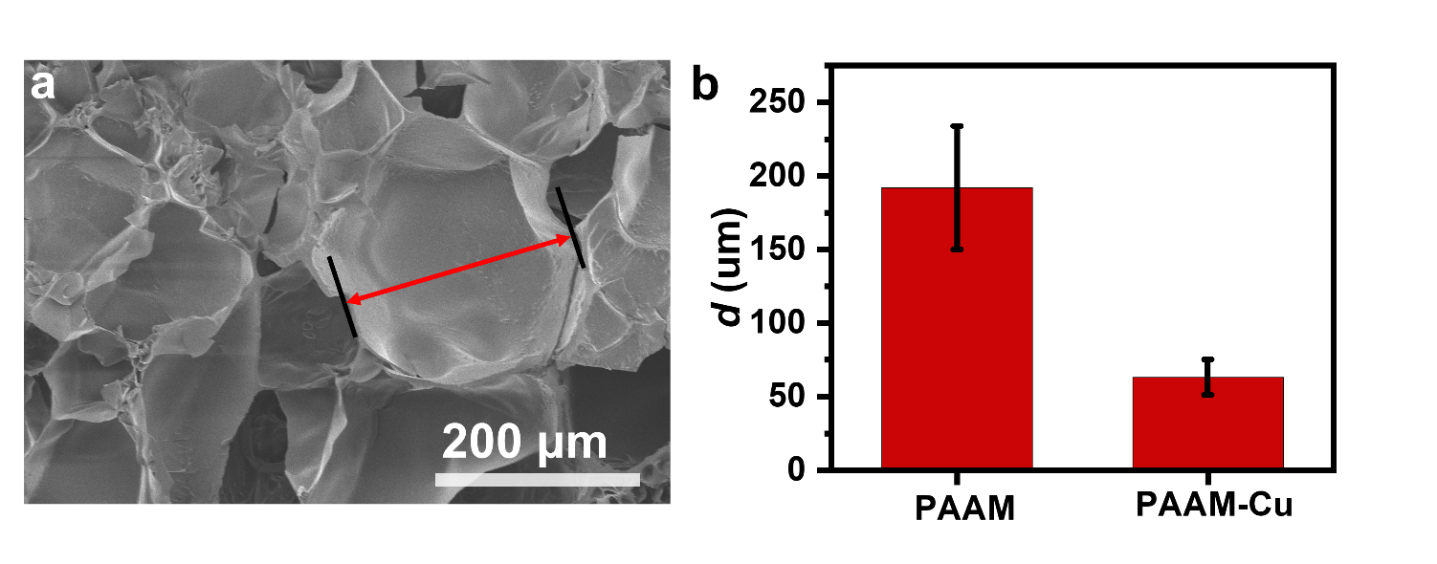


**Figure S3.** a) SEM image of PAAM xerogel b) Average pore size statistics of PAAM xerogel and PAAM-Cu xerogel.

**
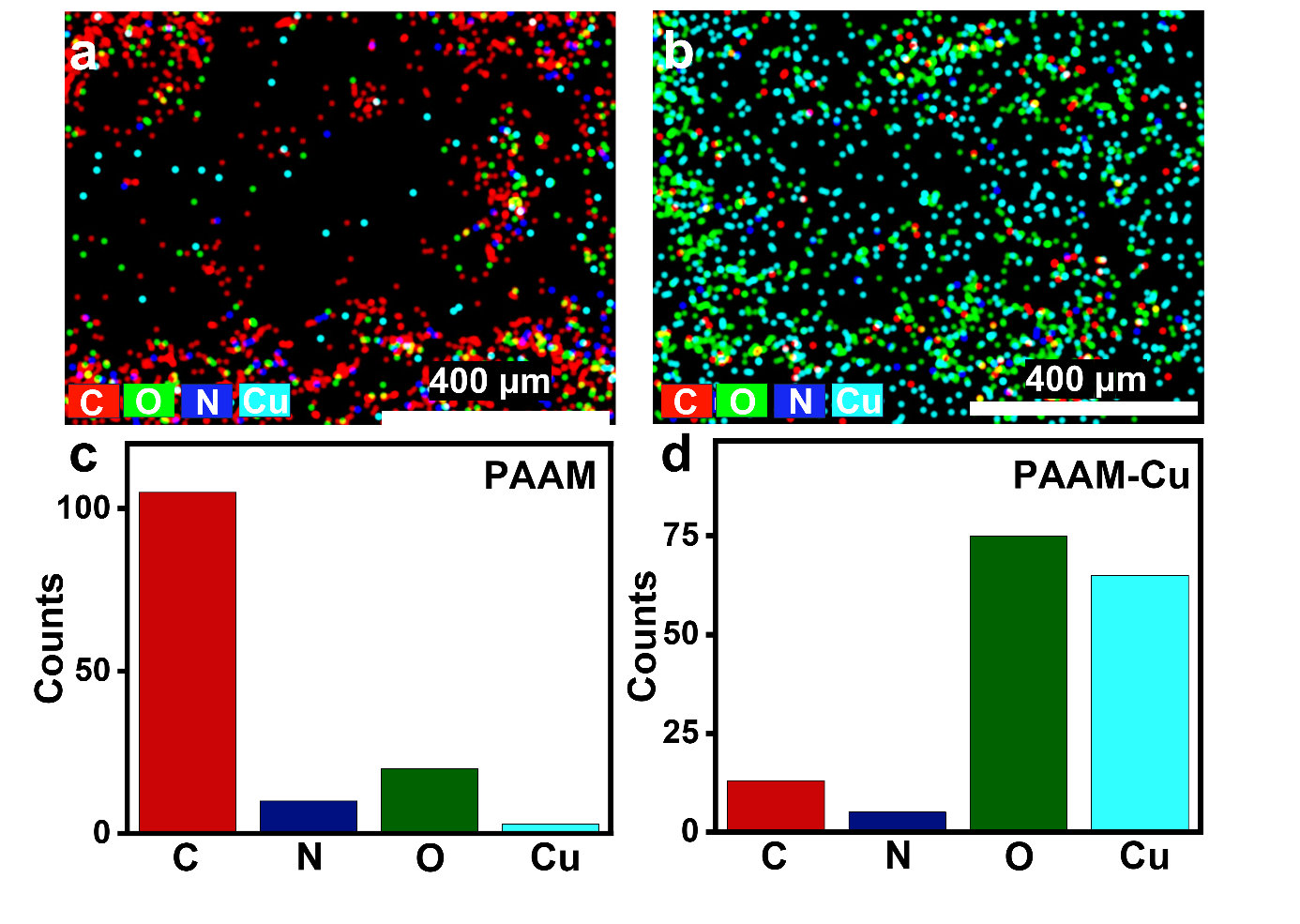
Figure S4.** Characterization of elemental distribution on the surface of PAAM and PAAM-Cu xerogels. a) EDS image of PAAM showing all elements. b) EDS image of PAAM-Cu showing all elements. c) Elemental distribution obtained from the EDS image of PAAM xerogel. d) Elemental distribution obtained from the EDS image of PAAM-Cu xerogel.


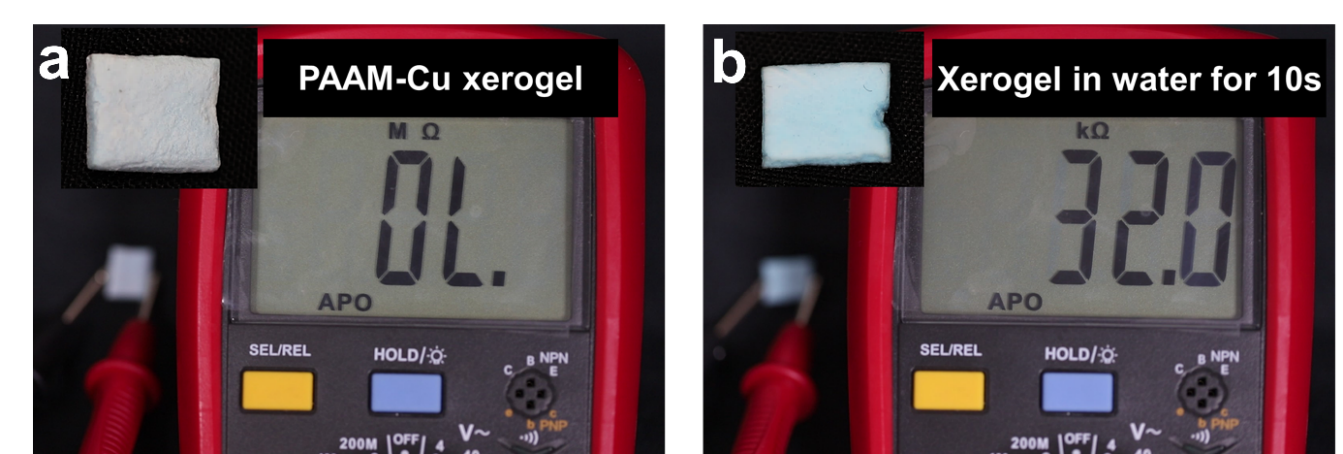


**Figure S5.** Resistance measurements of PAAM-Cu xerogel and hydrogel obtained by immersing the PAAM-Cu xerogel in water for 10 s, as measured using a multimeter. a) Resistance of the PAAM-Cu xerogel. b) Resistance of the hydrogel after immersing the PAAM-Cu xerogel in water for 10 s.


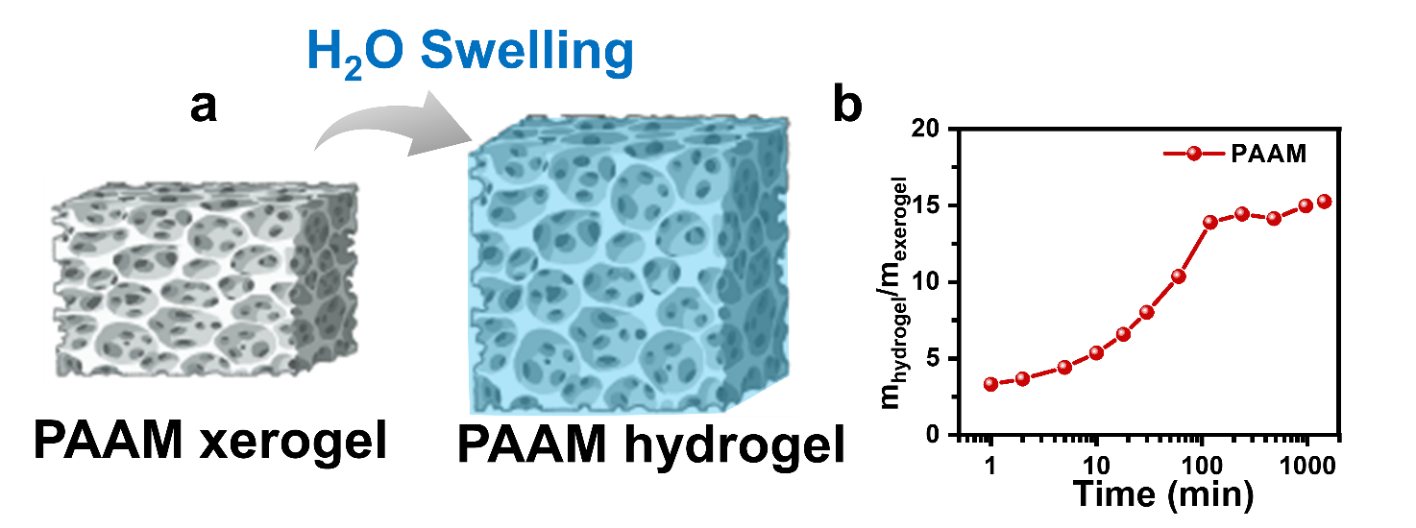


**Figure S6.** Mass ratio of PAAM xerogel during swelling in water. a) Schematic diagram illustrating the swelling of PAAM-xerogel into PAAM hydrogel. b) Mass ratio of the PAAM hydrogel to the PAAM-xerogel during the swelling process.

**Figure S7.** Electrical impedance spectroscopy and the fitted curve of PAAM-Cu hydrogels swollen in copper sulfate solutions of varying concentrations for 24 hours. The measurements were conducted using two gold-plated sheet electrodes, isolating the PAAM-Cu hydrogel.


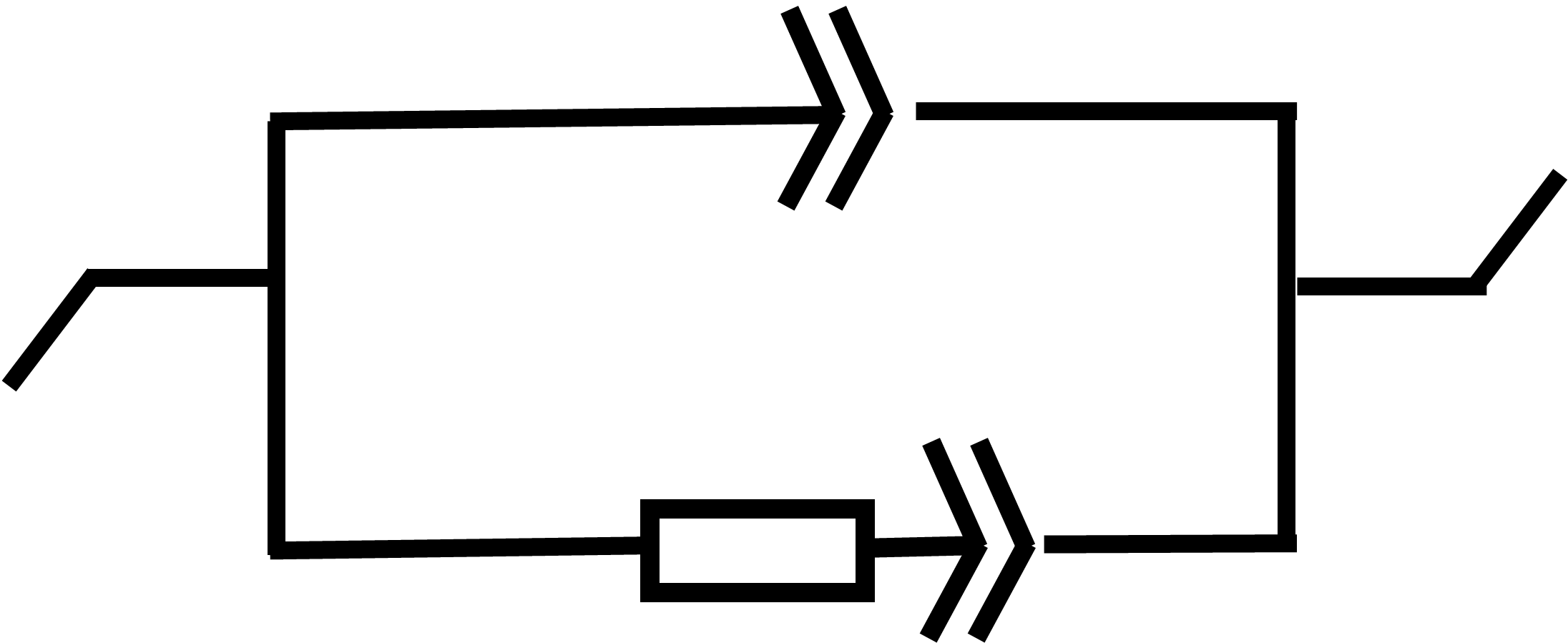


**Figure S8.** Equivalent circuit for the electrical impedance spectroscopy test


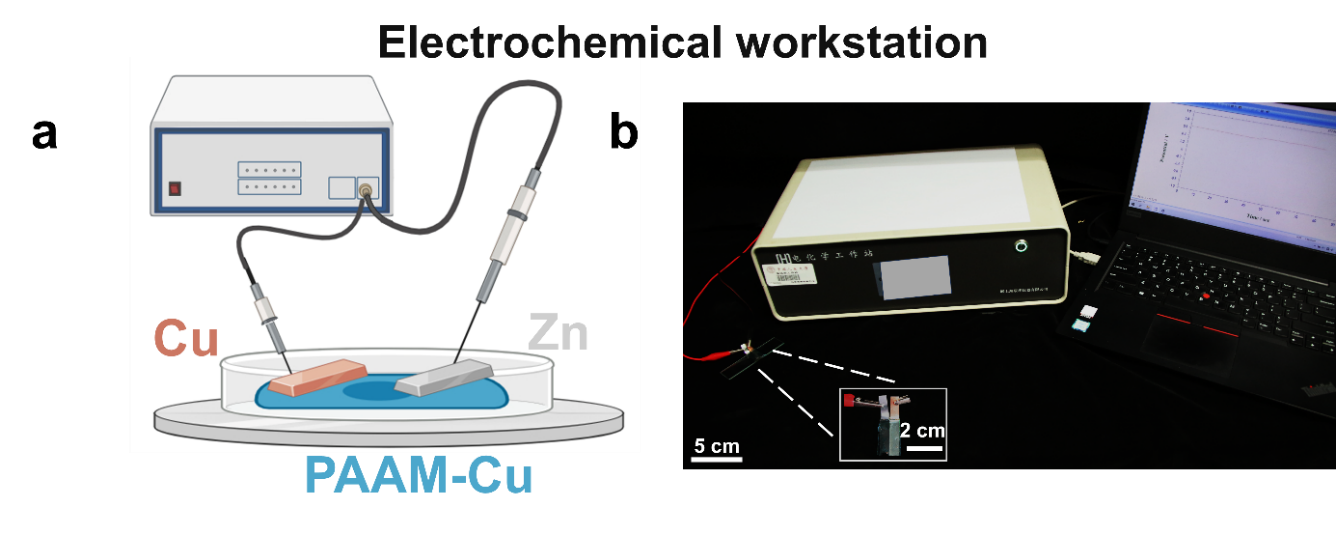


**Figure S9.** Electrical test instruments and test structure of the water-triggered battery unit. a) Schematic of the electrochemical workstation used for electrical testing. b) Photograph of the electrochemical workstation, with the test structure of the battery unit shown in the inset


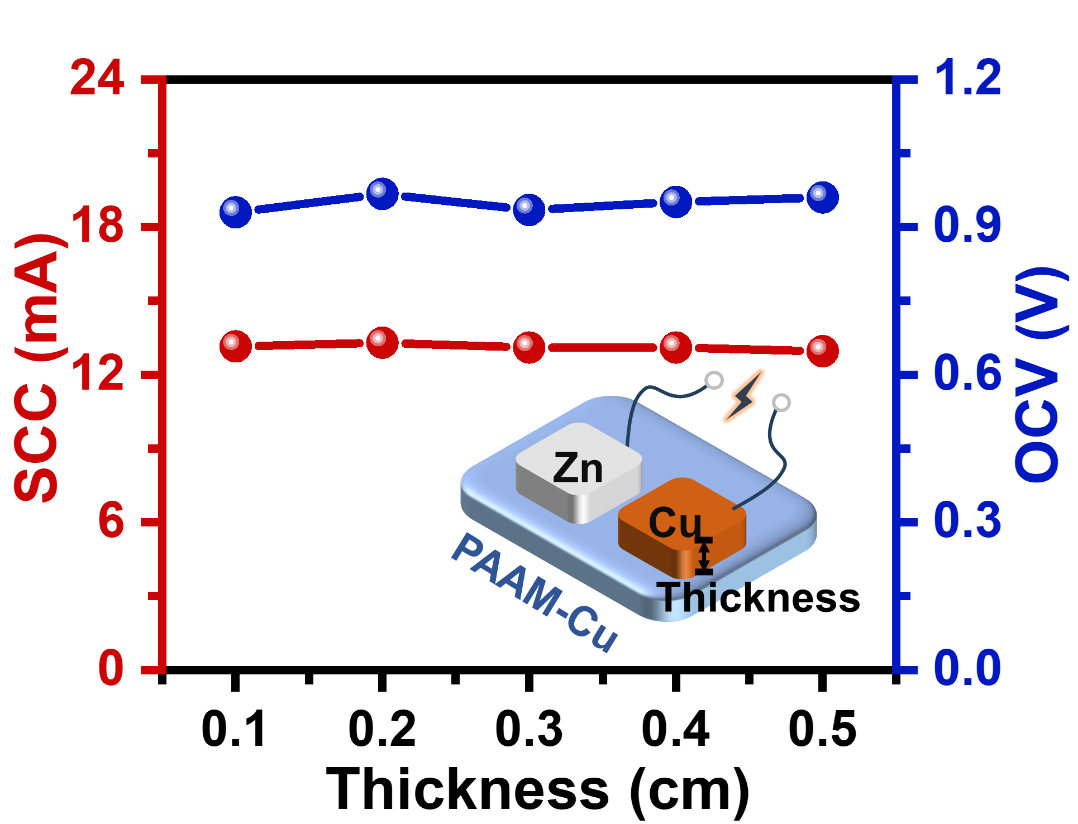


**Figure S10.** Open circuit voltage (OCV, blue ball) and short circuit current (SCC, red ball) of the battery unit with varying electrode sheet thicknesses.

**
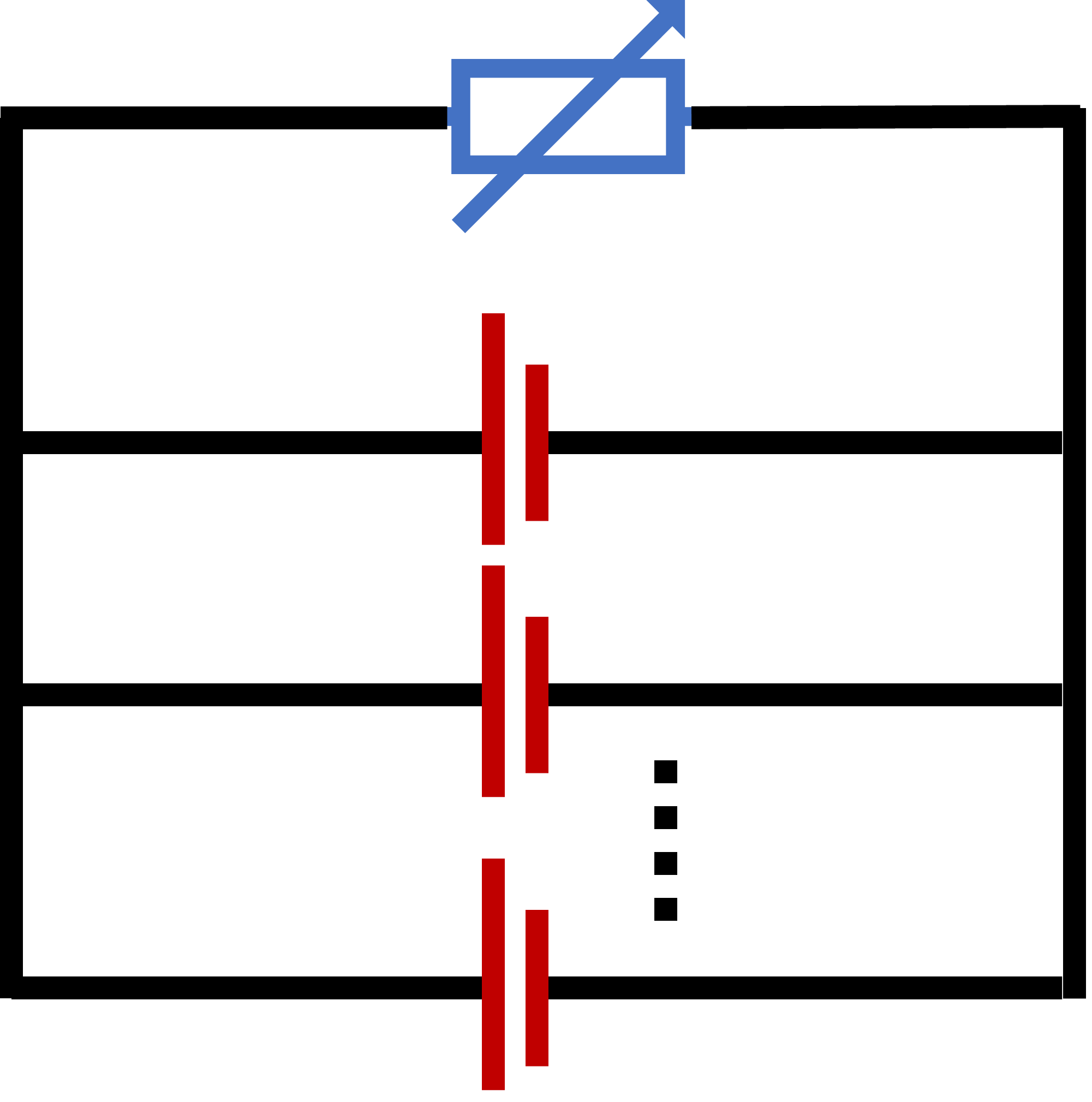
**

**Figure S11.** Equivalent circuit for electrodes of varying lengths. This schematic demonstrates that as the electrode length increases, additional batteries are connected in parallel, thereby increasing the current while maintaining a stable voltage.


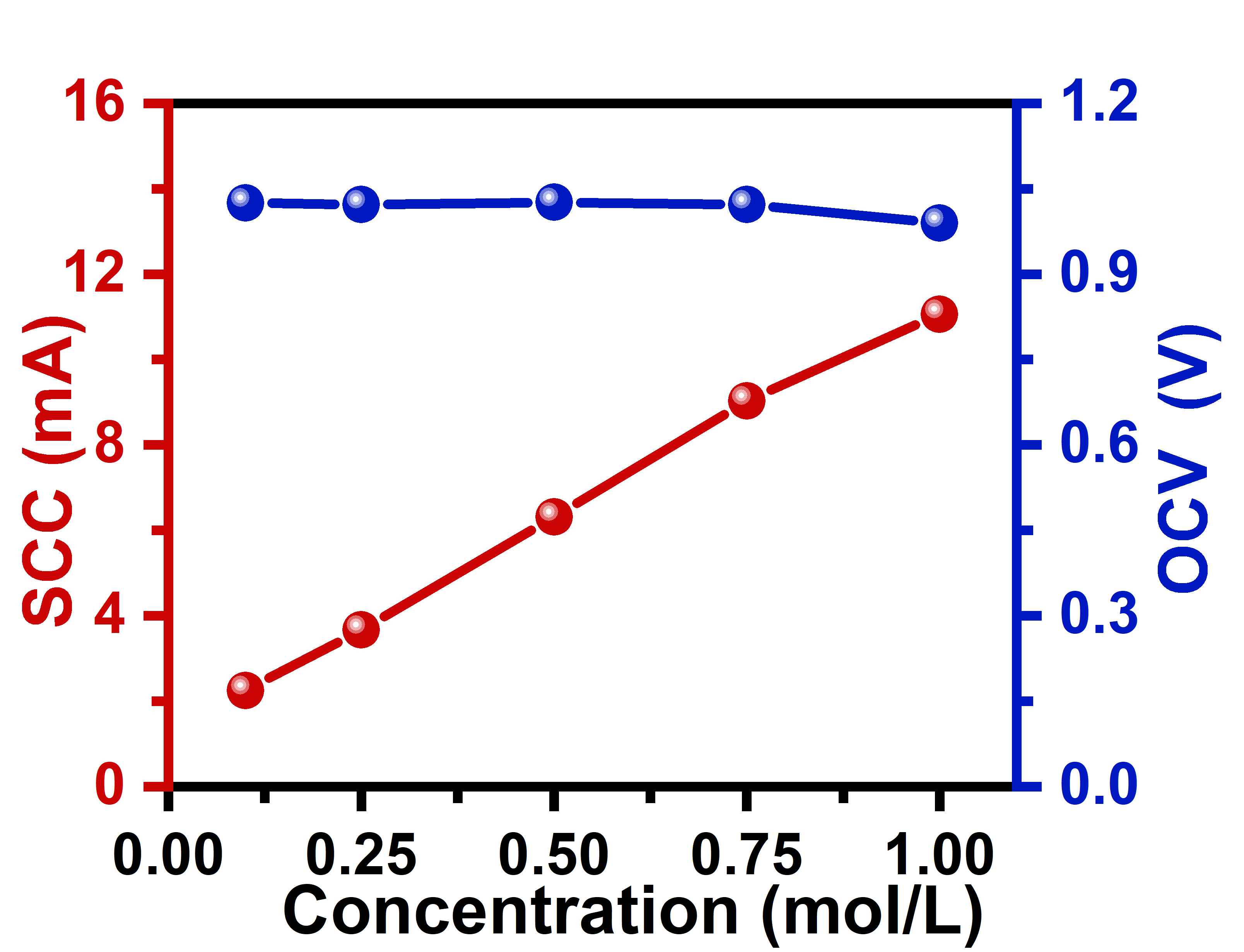


**Figure S12.** Open-circuit voltage (OCV, right) and short-circuit current (SCC, left) of the battery for the different concentrations of CuSO_4_ solution used for swelling the hydrogel.


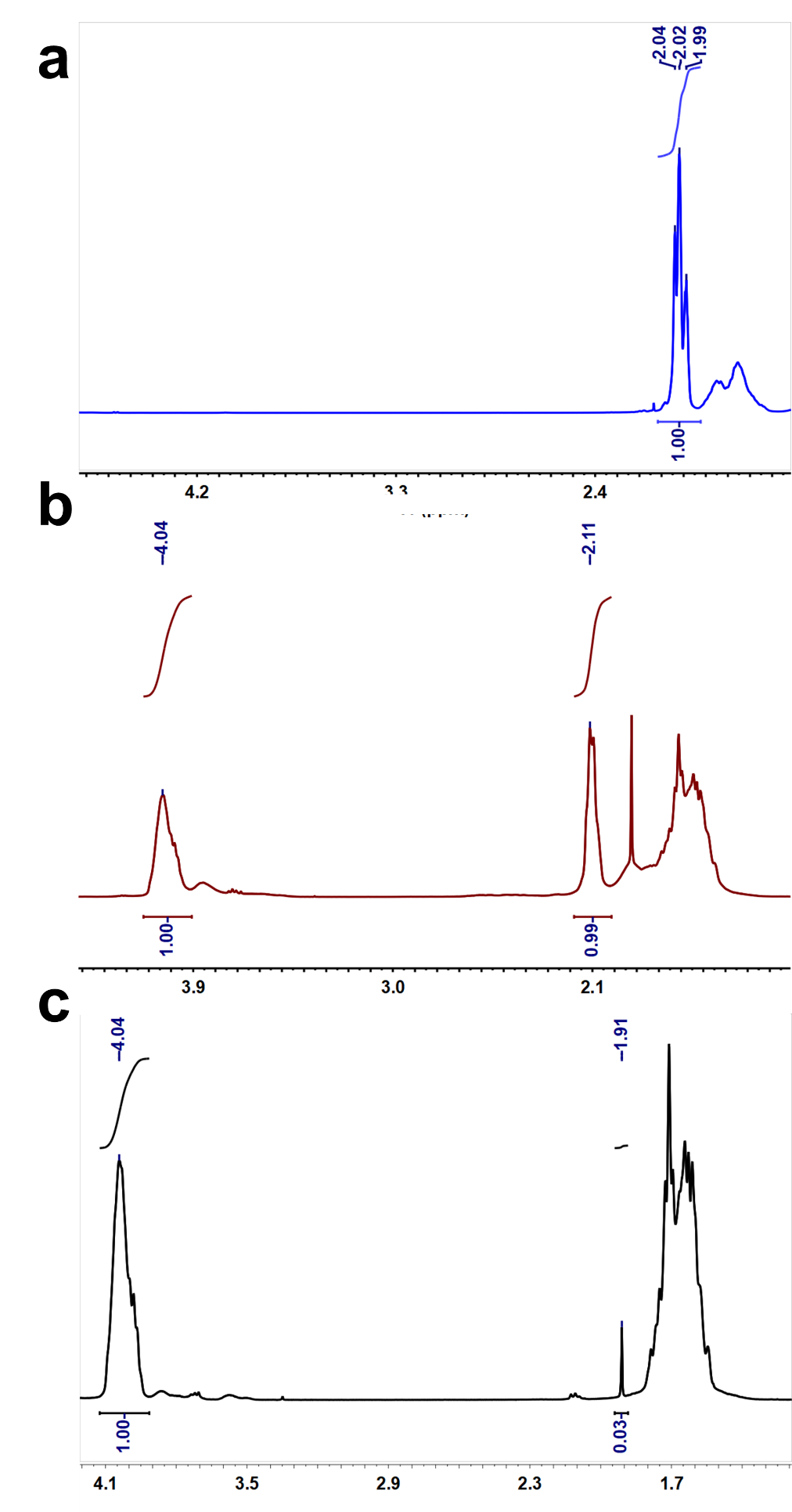


**Figure S13.** ^1^H NMR of spectra of the PVA-0 in D-chloroform (a), PVA-74 (b) and PVA-99(c) in D_2_O.

**
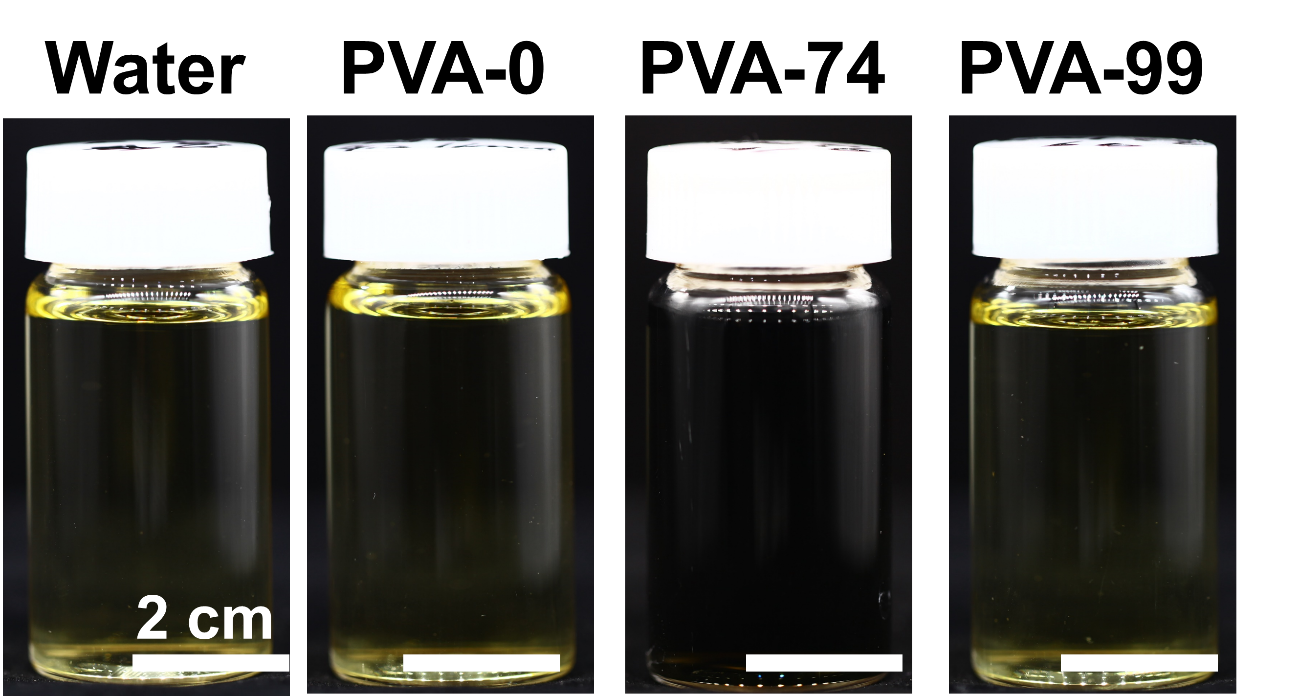
**

**Figure S14.** Photographs of the extracted solution of water and PVA films (PVA-0, PVA-74, and PVA-99) after dissolving in water for 1 min. The extracted solution was then treated with chromogens to display color development.


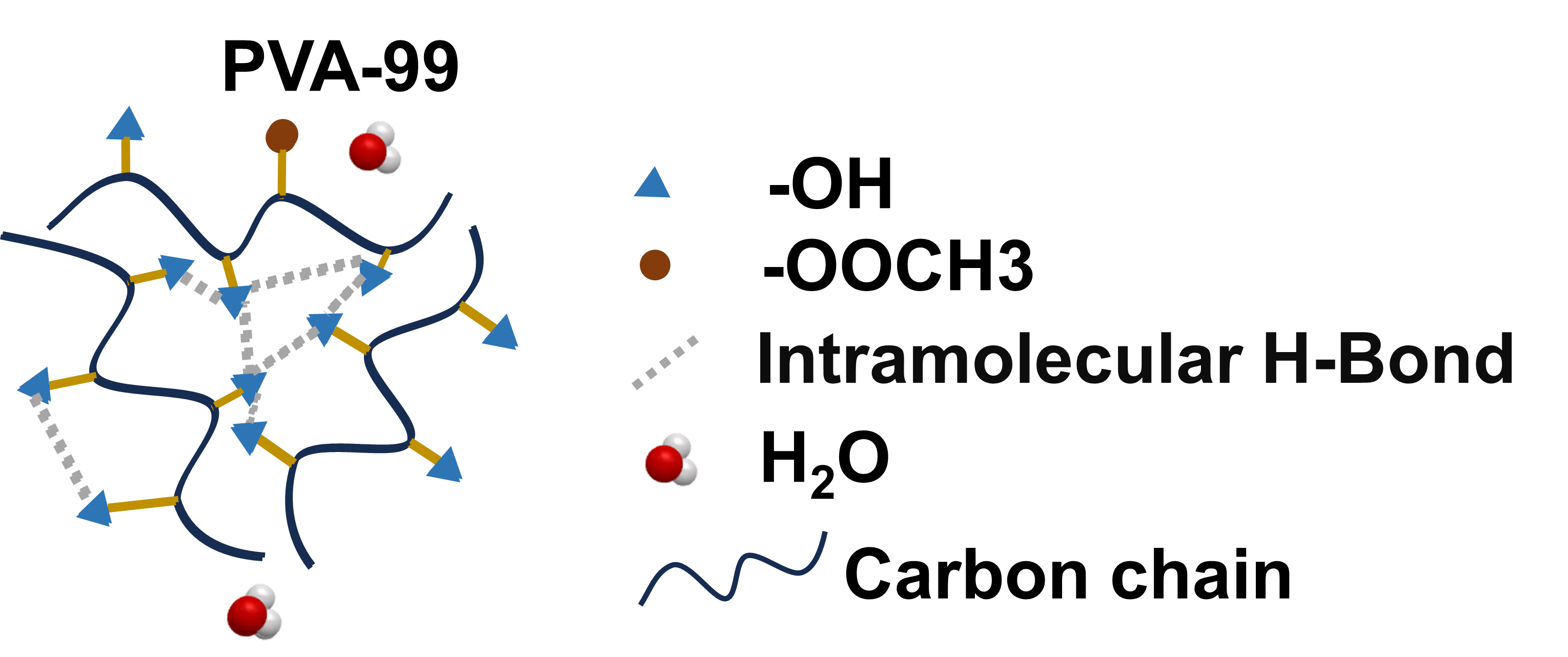


**Figure S15.** Schematic to illustrate the inability of PVA-99 film to dissolve in water.

**
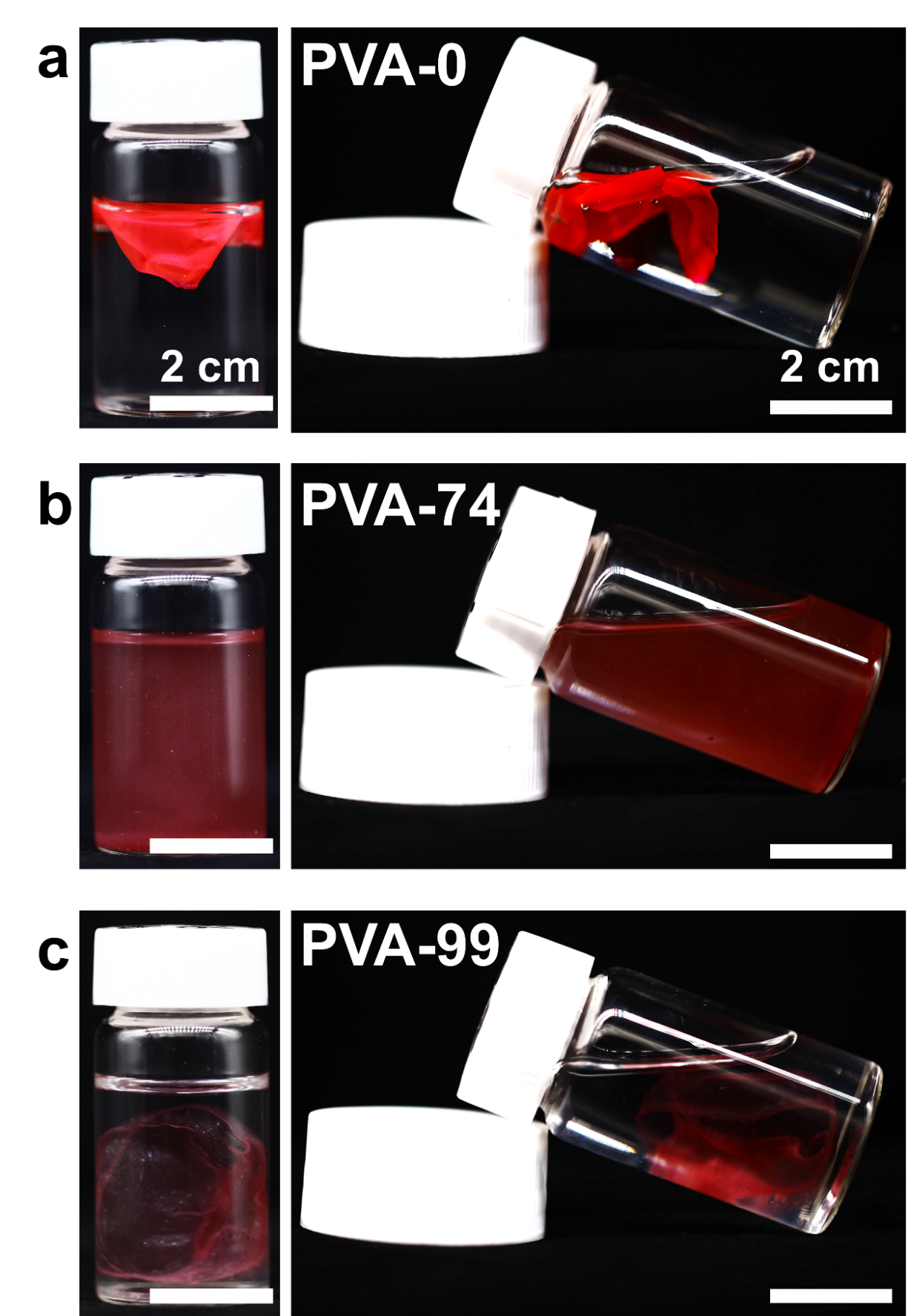
**

**Figure S16.** Water solubility of PVA films with varying degrees of alcoholysis. Photographs of PVA-0 (a), PVA-74 (b), and PVA-99 (c) films after being dissolved in water for 24 h, following Nile red staining


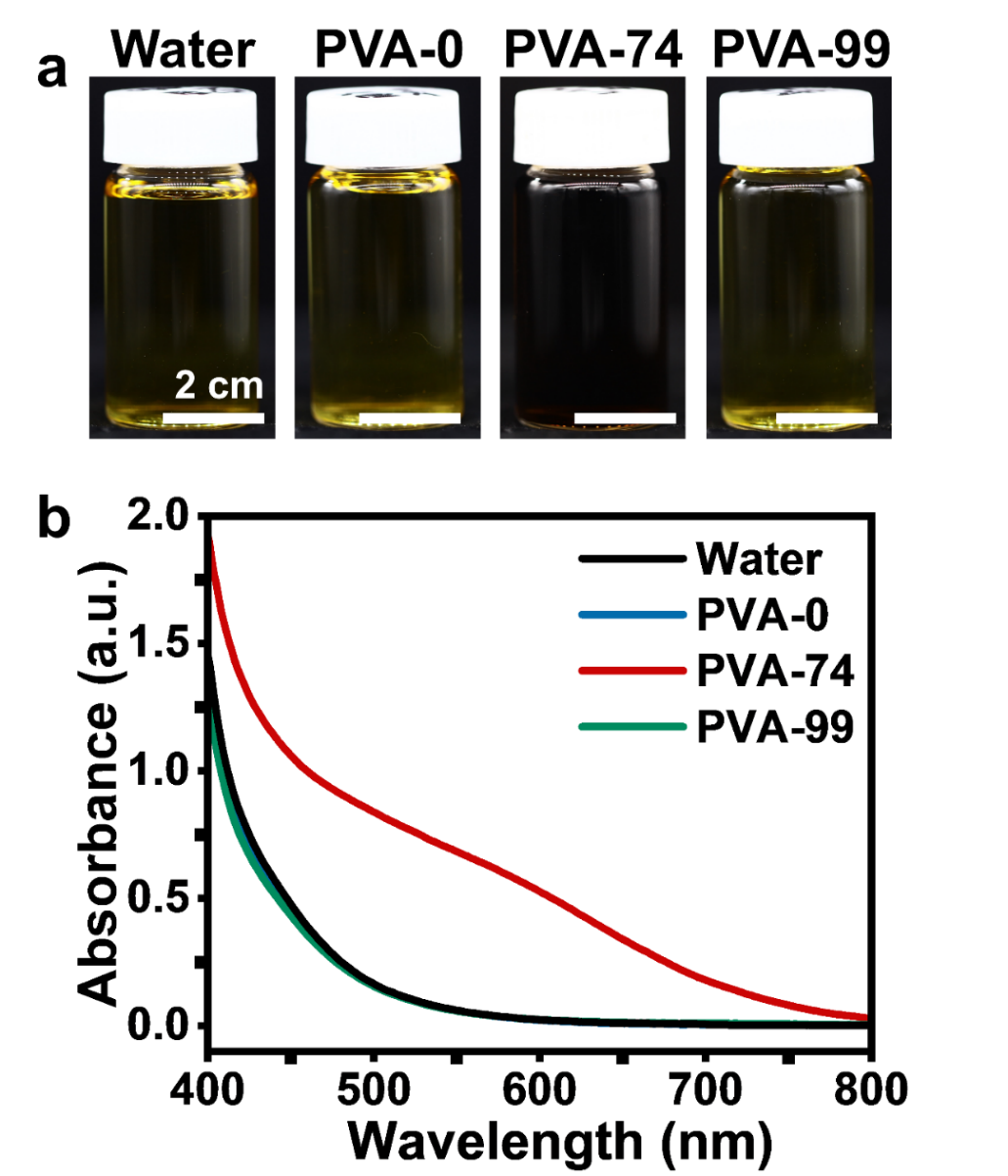


**Figure S17.** a) Photos of the extracted solution of water, the film of PVA-0, PVA-74, PVA-99 dissolved in water for 24 h and the color development with chromogens. b) UV-VIS spectra of water and the films of PVA-0, PVA-74, and PVA-99 dissolved in water after 1 h.


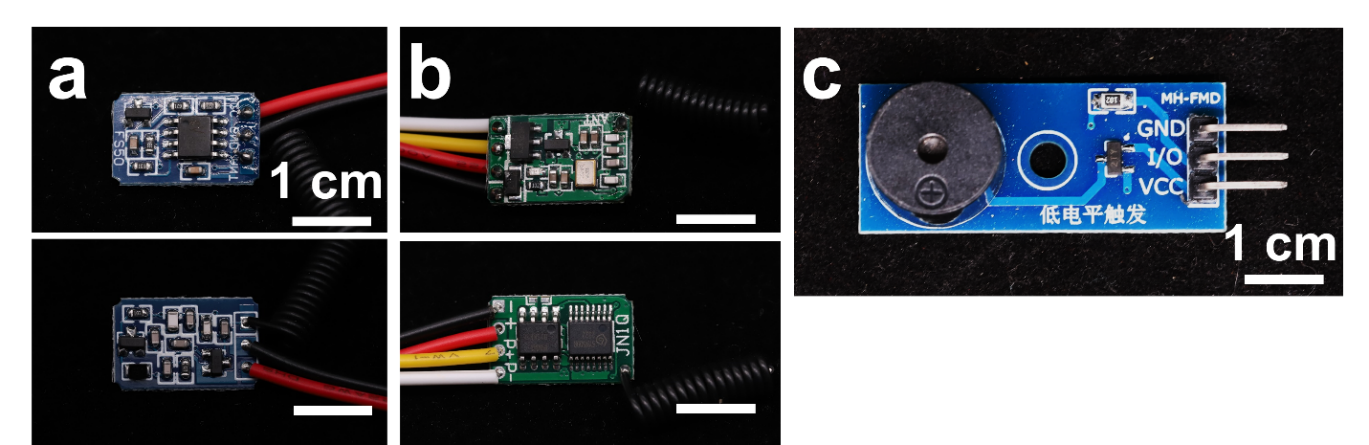


**Figure S18.** a) Photo of signal transmitter; b) Photo of signal receiver; c) Photo of buzzer.


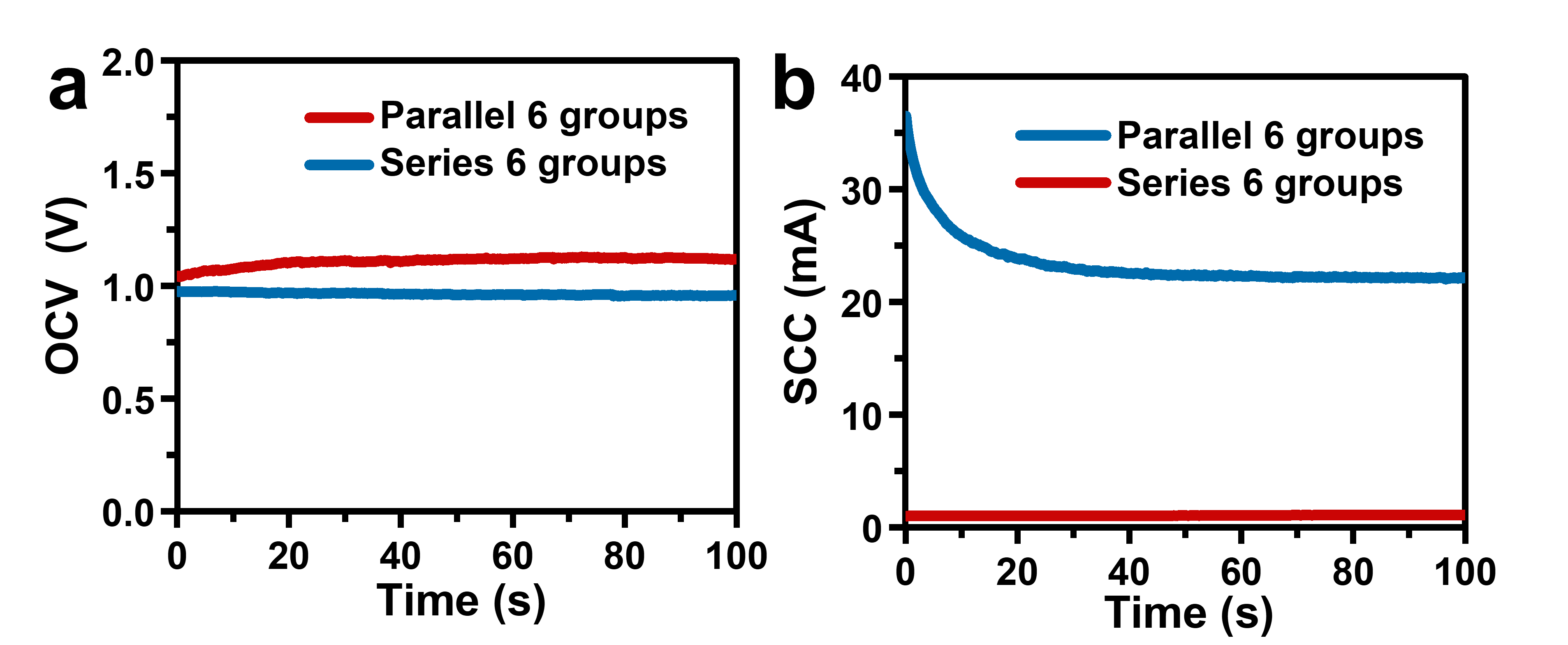


**Figure S19.** Comparison of the output from a single section of origami batteries in series and parallel configurations. a) Open-circuit voltage; b) Short-circuit current.


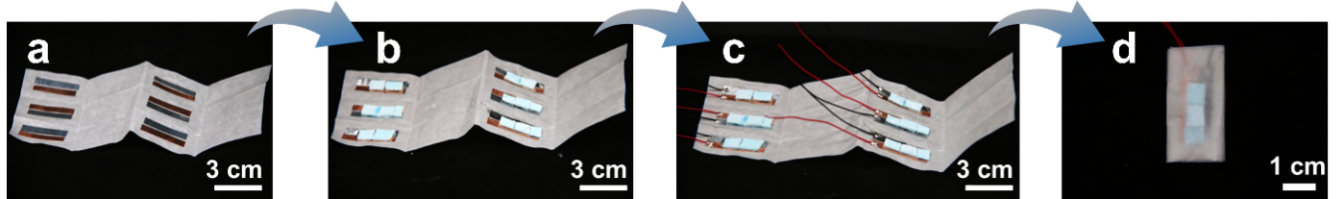


**Figure S20.** Origami battery connected in parallel preparation process. a) Six pairs of copper and zinc foils were glued to the paper substrate and the size of foil was 30.0 mm × 5.0 mm × 0.1 mm. b) PAAM-Cu xerogels were placed on the top of each electrode pair. c) The black wires were weld to the zinc foils and the red wires were weld to the copper foils. d) The battery array was folded through Miura origami into a small battery.

**
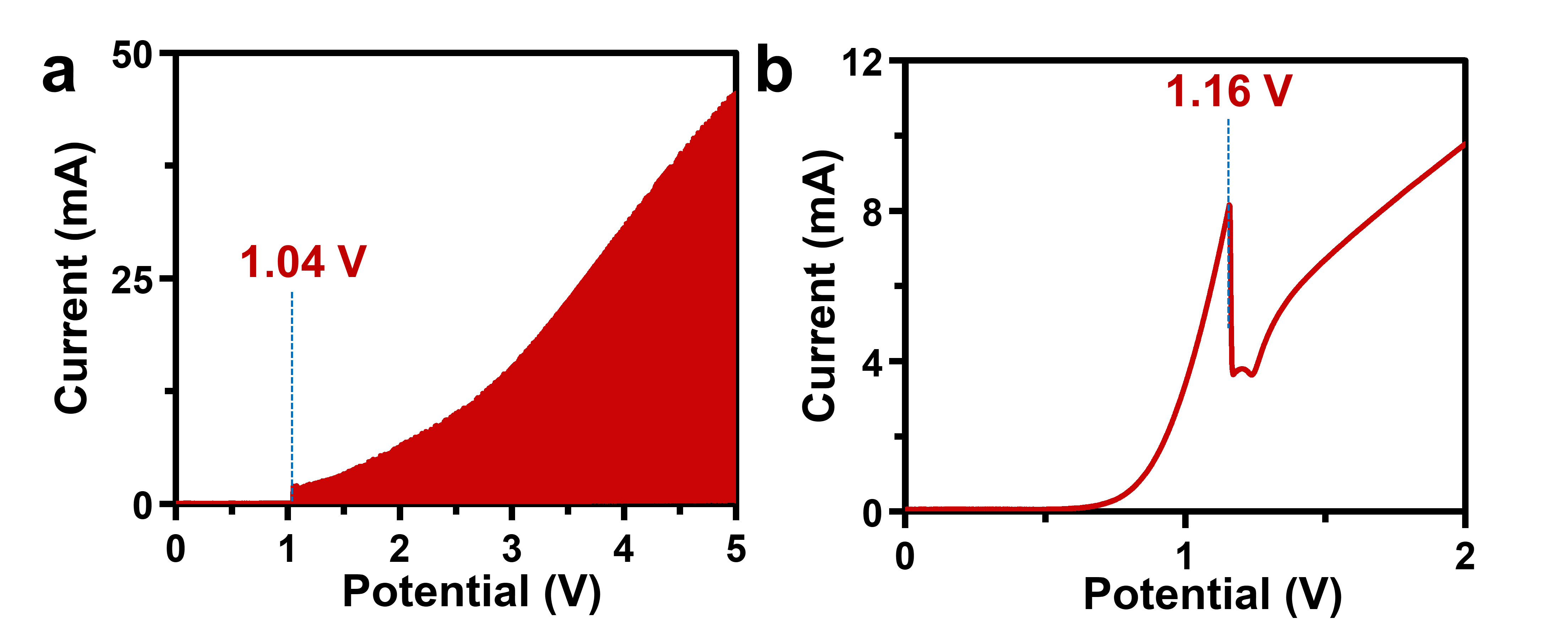
**

**Figure S21.** a) I-V curve of the signal transmitter. b) I-V curve of the buzzer.


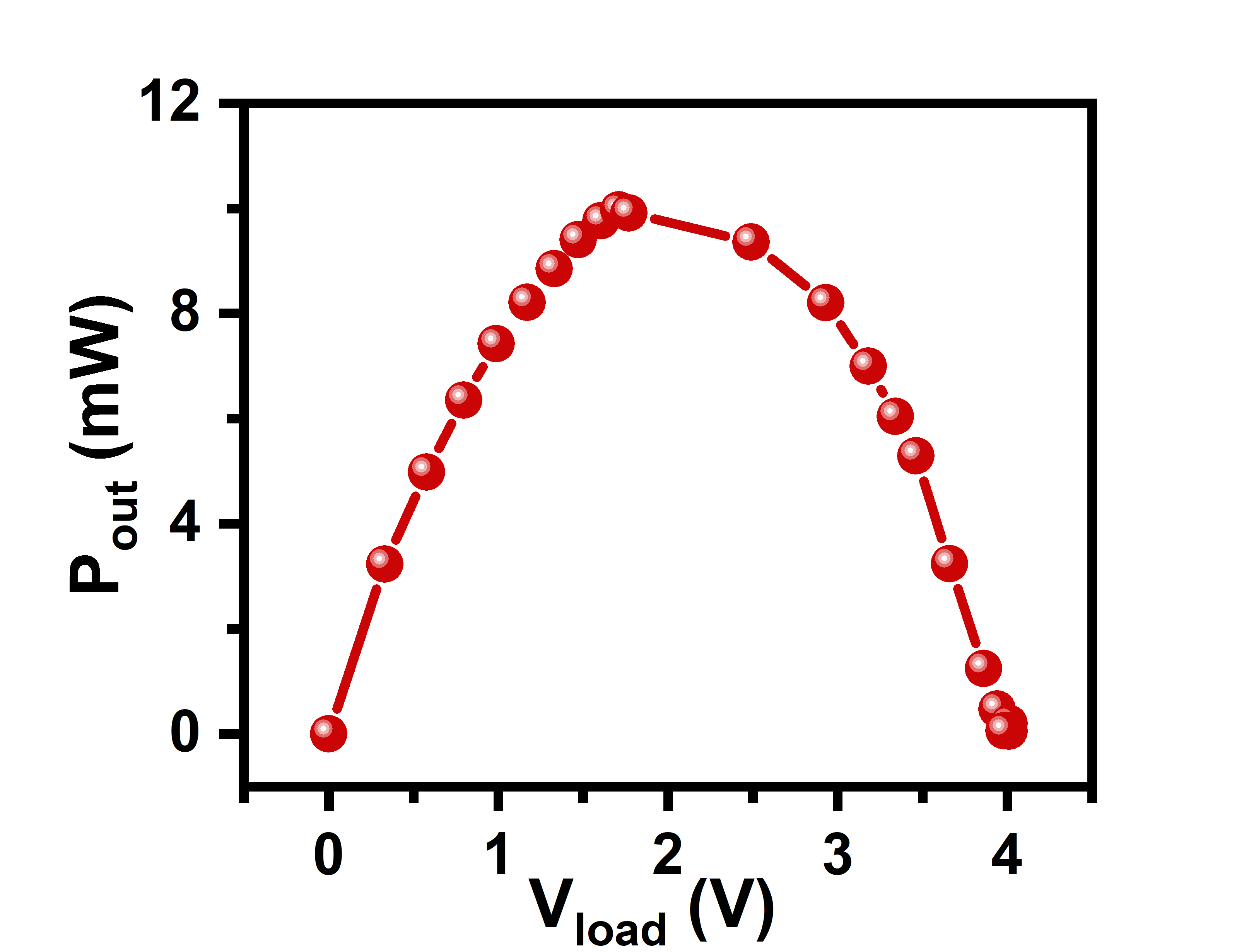


**Figure S22**. The output power of the integrated water-triggered batteries. To be specific, a resistor box was connected outside the circuit and the internal resistance of the resistor box was changed to test the generating power of the integrated batteries. The size of the integrated water-triggered batteries r is 4.5 cm x 2.0 cm x 0.6 cm and the surface area of the integrated batteries is 25.8 cm^2^.


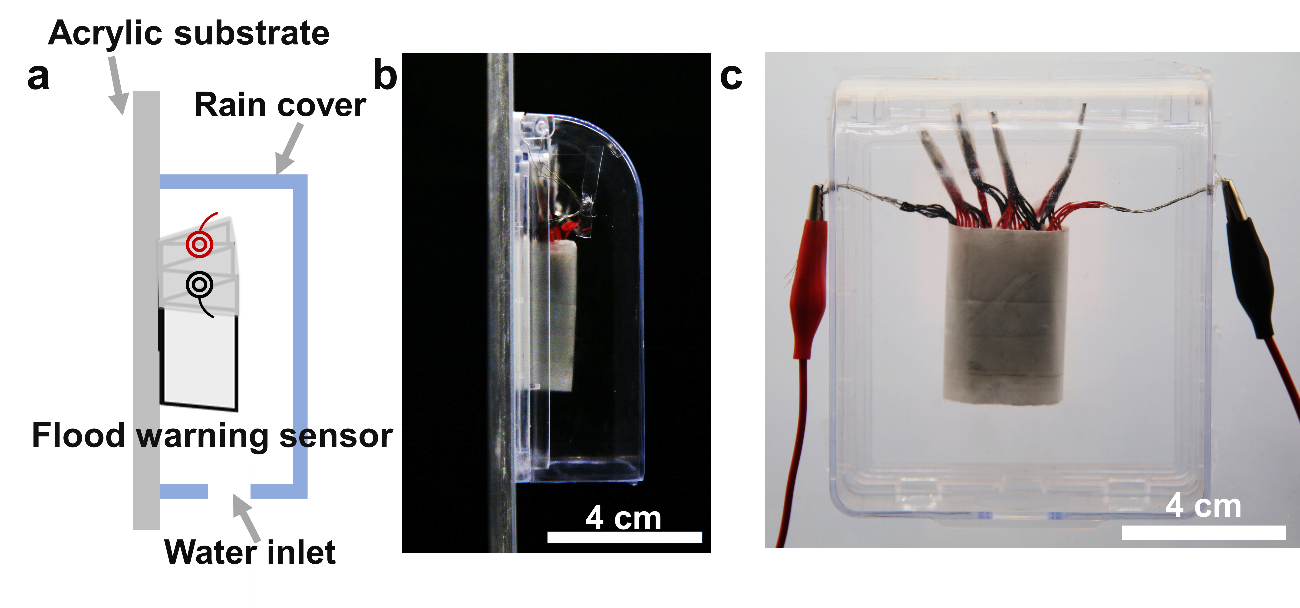


**Figure S23.** The integrated batteries combined with rain cover design to realize no false alarms in the event of rain. a) Design schematic. b) Side view. c) Front view of the integrated batteries.


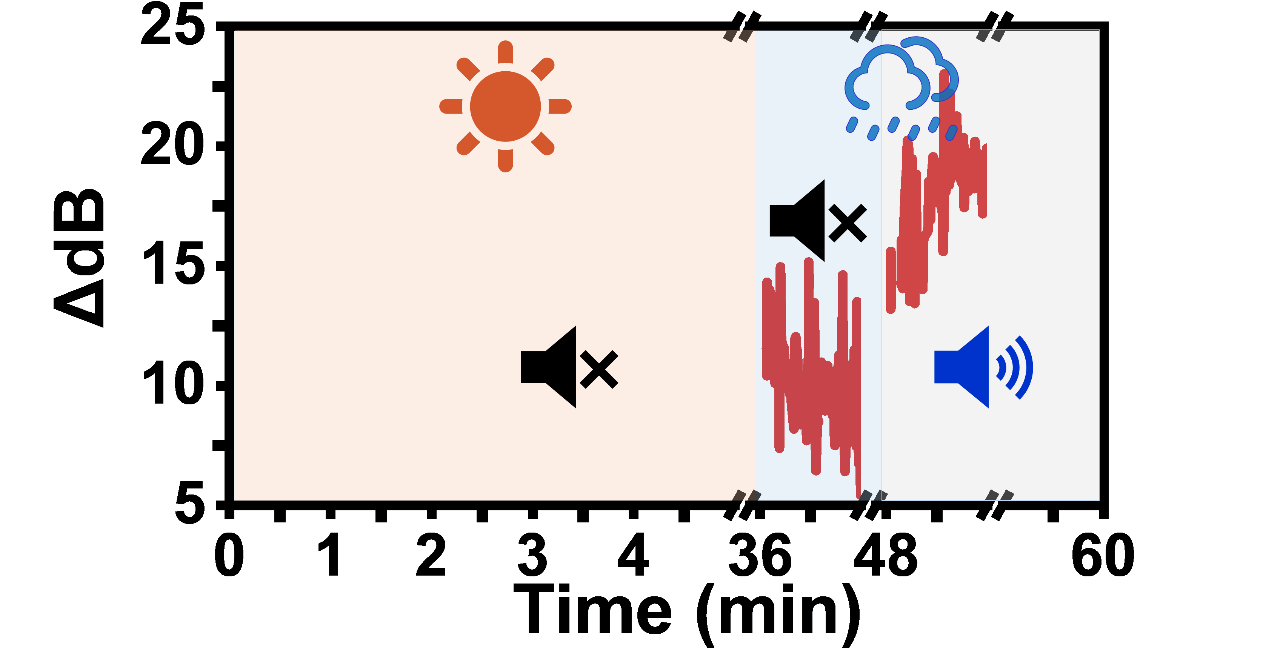


**Figure S24.** Plot of change in noise decibels in the last hour (using noise in the absence of rainfall as a baseline).


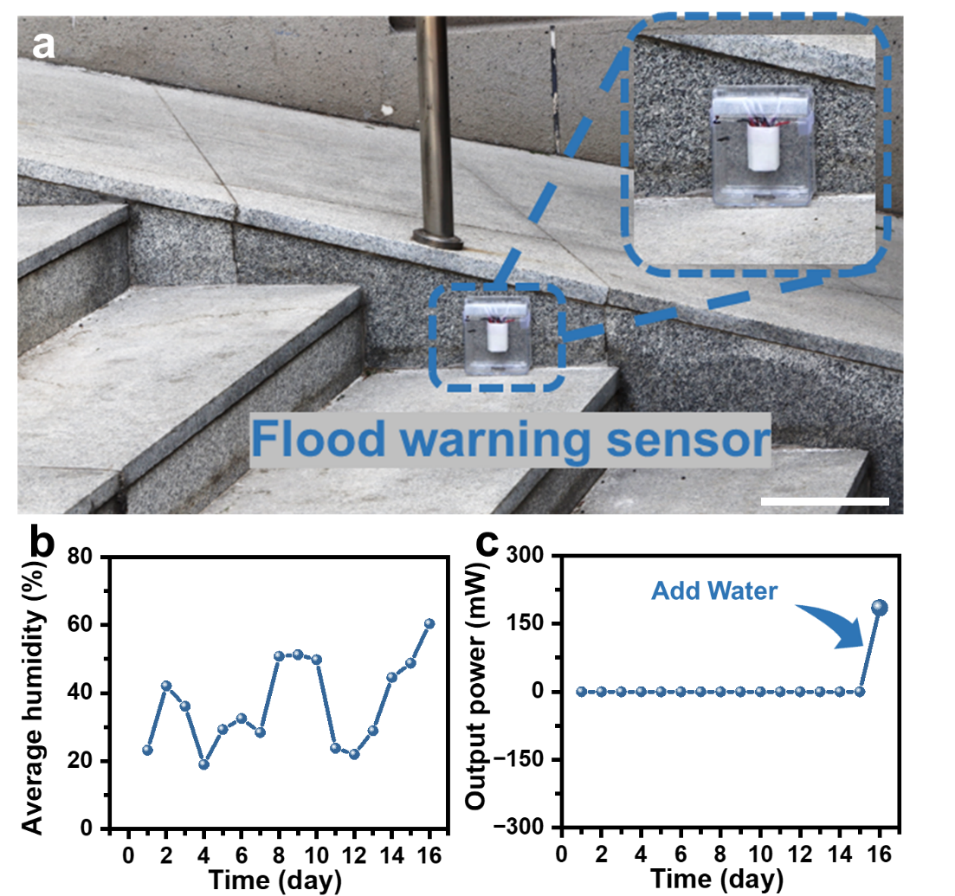


Figure S25. Long-term stability tests of the flood warning sensor. a) Deployment of the flood warning sensor outdoors. Scale bar: 20.0 cm. b) Average humidity recorded outdoors for two weeks. c) The electrical output powers tested on the sensor outdoors for two weeks.


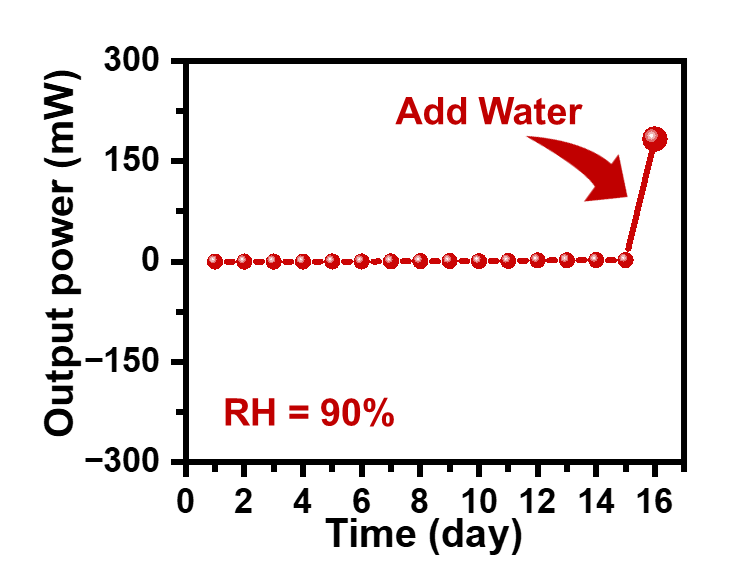


Figure S26.The electrical output powers tested on the sensors under 90% relative humidity conditions for two weeks.
